# Determination of Dynamic Brain Connectivity via Spectral Analysis

**DOI:** 10.3389/fnhum.2021.655576

**Published:** 2021-07-16

**Authors:** Peter A. Robinson, James A. Henderson, Natasha C. Gabay, Kevin M. Aquino, Tara Babaie-Janvier, Xiao Gao

**Affiliations:** ^1^School of Physics, University of Sydney, Sydney, NSW, Australia; ^2^Center of Excellence for Integrative Brain Function, University of Sydney, Sydney, NSW, Australia; ^3^Department of Biomedical Engineering, University of Melbourne, Parkville, VIC, Australia

**Keywords:** brain connectivity, neural field theory, effective connectivity, functional connectivity, modeling

## Abstract

Spectral analysis based on neural field theory is used to analyze dynamic connectivity via methods based on the physical eigenmodes that are the building blocks of brain dynamics. These approaches integrate over space instead of averaging over time and thereby greatly reduce or remove the temporal averaging effects, windowing artifacts, and noise at fine spatial scales that have bedeviled the analysis of dynamical functional connectivity (FC). The dependences of FC on dynamics at various timescales, and on windowing, are clarified and the results are demonstrated on simple test cases, demonstrating how modes provide directly interpretable insights that can be related to brain structure and function. It is shown that FC is dynamic even when the brain structure and effective connectivity are fixed, and that the observed patterns of FC are dominated by relatively few eigenmodes. Common artifacts introduced by statistical analyses that do not incorporate the physical nature of the brain are discussed and it is shown that these are avoided by spectral analysis using eigenmodes. Unlike most published artificially discretized “resting state networks” and other statistically-derived patterns, eigenmodes overlap, with every mode extending across the whole brain and every region participating in every mode—just like the vibrations that give rise to notes of a musical instrument. Despite this, modes are independent and do not interact in the linear limit. It is argued that for many purposes the intrinsic limitations of covariance-based FC instead favor the alternative of tracking eigenmode coefficients vs. time, which provide a compact representation that is directly related to biophysical brain dynamics.

## 1. Introduction

Brain activity spans many decades of spatial and temporal scale and constantly changes due to stimuli and internally generated signals (Raichle, 2011). Some of these changes are due to changed activity (e.g., evoked by stimuli) in the pre-existing brain structure, while others are due to changes in this structure that alter the activity—i.e., changes in neural connections and their strengths (Bassett et al., 2011, 2017; Deco et al., 2011; Raichle, 2011; Hutchison et al., 2013a,b; Calhoun et al., 2014; Kopell et al., 2014; Preti et al., 2017; Babaie-Janvier and Robinson, 2019, 2020). It is thus of central interest to determine how the latter dynamic readjustments of connectivity are caused by, and support, signal processing demands placed on the brain during tasks.

On learning and developmental timescales of 100 s or more, synaptic connections can be formed and broken and long-term plasticity plays a key role in optimizing brain responses (Koch, 1999; Ghosh et al., 2008; Bassett et al., 2011; Kopell et al., 2014), as do neuromodulatory effects during cognitive processes and the sleep-wake cycle (Koch, 1999; Tagliazucchi and Laufs, 2014). Effective connectivity (EC) is defined to be the strength of connections, direct or indirect, between two points. On timescales below a few seconds, changes in EC due to modification of synaptic strengths, firing thresholds, and other aspects of neural responsiveness can result from a range of biophysical processes such as adaptation and facilitation (Koch, 1999; Rennie et al., 1999, 2002; Robinson and Roy, 2015), and these affect EEG activity and evoked responses (Rennie et al., 1999; Babaie-Janvier and Robinson, 2019, 2020). There the change in connectivity can be interpreted as part of the response that implements attention (Babaie-Janvier and Robinson, 2019, 2020). In the intermediate range of roughly 5–100 s, processes such as plasticity certainly exist that can change functional brain connectivity, but their role is less understood.

What is perhaps more puzzling than the occurrence of temporal changes *per se* is that these often appear to be quite rapid and widespread (Britz et al., 2010; Cabral et al., 2014; Hansen et al., 2015; Preti et al., 2017; Michel and Koenig, 2018). Much of the evidence for such changes is from studies of functional connectivity (FC), which is most commonly defined to be the covariance of activity at pairs of points and is often described in terms of patterns termed resting state “networks” (Friston, 1994, 2011; Raichle et al., 2001; Bullmore and Sporns, 2009; Sporns, 2011; Zalesky et al., 2012; Hutchison et al., 2013a; Zalesky and Breakspear, 2015; Fornito et al., 2016). The basis for this definition is the assertion that positively correlated points are likely to be involved in supporting the same function, an issue to which we return later where we show that negative correlations are equally important to the dynamics (Robinson, 2019). Because FC is defined in terms of activity, it is quite possible for it to change quickly and large-scale activity patterns are observed to change on timescales as short as 50–100 ms using EEG and MEG (Britz et al., 2010; Musso et al., 2010; Van De Ville et al., 2010; Michel and Koenig, 2018), or over tens of seconds using functional MRI (fMRI) (v et al., 1995; Damoiseaux et al., 2006; Fox and Raichle, 2007; Britz et al., 2010; Chang and Glover, 2010; Deco and Jirsa, 2012; Hipp et al., 2012; Hutchison et al., 2013a,b; Calhoun et al., 2014; Mitra et al., 2014; Hansen et al., 2015; Chang et al., 2016; Cabral et al., 2017; Babaie-Janvier and Robinson, 2019; Hunyadi et al., 2019). Notably, the patterns of activity and inferred connectivity are similar, but not identical, across different states of arousal, under both spontaneous and task-based conditions, and when observed using differing measurement modalities (Peltier et al., 2005; Smith et al., 2009; Tagliazucchi and Laufs, 2014; Chang et al., 2016; Robinson, 2019).

Rapid large-scale reorganizations of FC have been reported by numerous authors working with fMRI, particularly when matching to libraries of patterns is used in its quantification (Deco et al., 2011, 2017; Calhoun et al., 2014; Hansen et al., 2015; Cabral et al., 2017). This is at least in part because matching is done moment by moment to the most similar of a finite number of patterns, which necessarily induces apparent rapid interstate jumps or “switching” even if the actual underlying dynamics are smooth. Similar sudden jumps between EEG microstates (which are characteristic large-scale patterns of scalp potential) have been asserted when a small number of microstates are used for matching (Britz et al., 2010; Gabay et al., 2018). However, the latter effect has been shown to be consistent with smooth evolution (no jumps) being projected onto the nearest discrete microstate at each point in time (Gabay et al., 2018). It would thus be advantageous to develop methods that allow for continuous evolution, with sufficient time resolution to follow rapid changes when and if they occur. The ability to discriminate, if possible, between effects of changing structure and changing activity on FC would also be valuable.

One limitation on tracking dynamic FC is that it is most often measured using fMRI, which is slow and usually involves calculating the covariance within a sliding window of tens of seconds in duration (Fox and Raichle, 2007; Bullmore and Sporns, 2009; Chang and Glover, 2010; Deco et al., 2011; Hutchison et al., 2013a,b; Allen et al., 2014; Leonardi et al., 2015; Chang et al., 2016; Hindriks et al., 2016). This limits temporal resolution and introduces artifacts due to contributions from fluctuations with periods longer than the window length (Yule, 1926; Leonardi et al., 2015; Zalesky and Breakspear, 2015; Ernst et al., 2017). This has resulted in a host of attempts to quantify FC changes and to separate true changes from windowing artifacts. These have overwhelmingly been statistically based and have involved many disjoint methods, including correlations of FC with EC or structural connectivity (SC; i.e., anatomical connectivity), abstract graph theory, clustering, PCA, ICA, matching to dictionaries of predetermined patterns and many others (Arfanakis et al., 2000; Beckmann et al., 2005; Bullmore and Sporns, 2009; Greicius et al., 2009; Honey et al., 2009, 2010; Smith et al., 2009; Pernice et al., 2011; Yeo et al., 2011; Fornito et al., 2013; Leonardi et al., 2013, 2014; Allen et al., 2014; Anderson et al., 2014; Calhoun et al., 2014; Mitra et al., 2014; Hansen et al., 2015; Yaesoubi et al., 2015; Eklund et al., 2016; Cabral et al., 2017; Preti and Van De Ville, 2019), as reviewed by a number of authors (Deco et al., 2011; Sporns, 2011; Fornito et al., 2016; Bassett et al., 2017; Preti et al., 2017). As well as many methods being ad hoc and/or assuming particular connectivity architectures such as modularity or hierarchy, the relationships between different methods and their respective results are often obscure or unknown.

**Figure 1:**
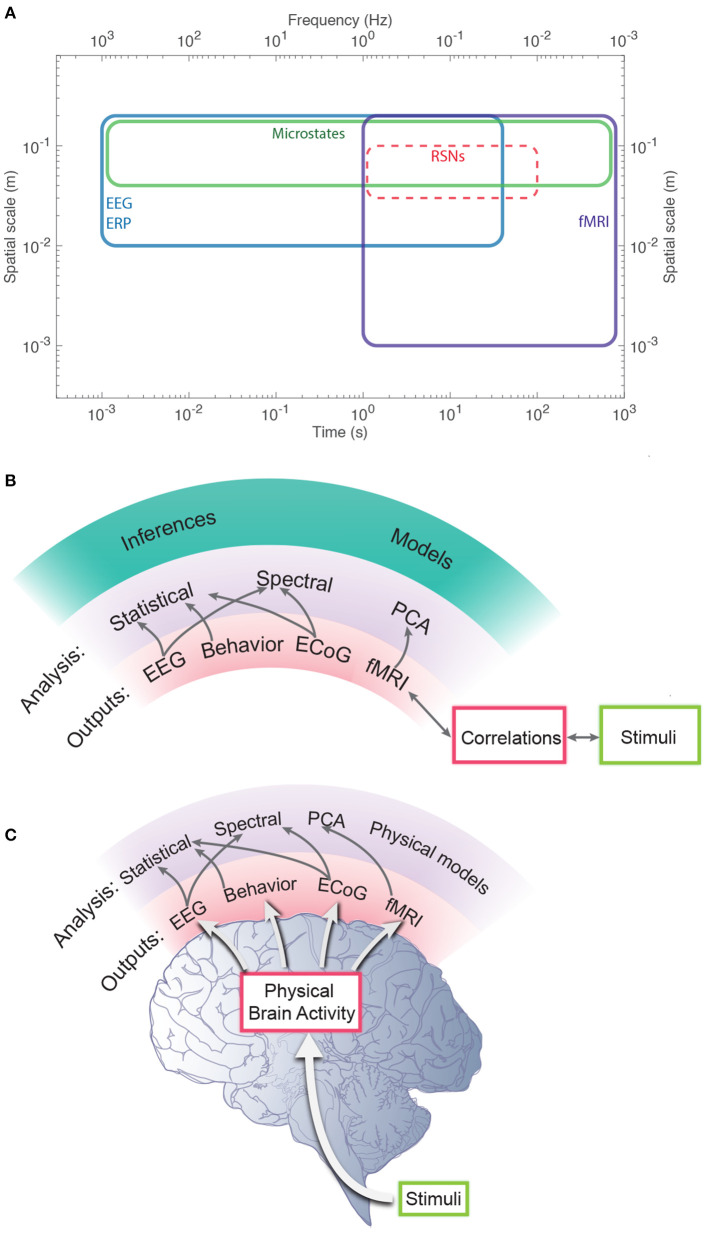
Schematic of brain observations and analysis. **(A)** Approximate spatiotemporal scales spanned by EEG, ERP, and fMRI observations, and by phenomenological resting state networks and microstates. **(B)** Phenomenological and statistical links between measurements and constructs. **(C)** Physical activity generates the links seen in **(B)**.

We stress that phenomenological and statistical methods often yield robust differences between conditions, and can be used to narrow the range of possible links between phenomena and for classification and hypothesis testing, but their nature prevents them from providing direct links to physical mechanisms. In contrast, physically based models have increasingly exposed these links in a straightforward way, showing how EC supports activity, and how correlations and resulting FC can be calculated directly from EC (Robinson, 2012, 2019; Friston et al., 2014; Robinson et al., 2014, 2016). It is found that the total EC is equivalent to the system transfer function that relates outputs to inputs (Robinson, 2012, 2019), thereby linking such analyses directly to engineering control systems (Babaie-Janvier and Robinson, 2018, 2019, 2020). Notably, such approaches explicitly link changes in EC and/or activity with resulting FC changes, which can be especially rapid when caused by changes in activity rather than structure or gain. Moreover, work in the last decade has shown that the inverse problem of determining EC from FC can be solved in a wide variety of situations (Galán, 2008; Friston et al., 2014; Robinson et al., 2014, 2016; MacLaurin and Robinson, 2019; Robinson, 2019; Henderson et al., 2021), as can the problem of obtaining EC, and hence FC, from evoked responses (Robinson et al., 2018; Henderson et al., 2021). Figure 1 shows approximate scales spanned by EEG, fMRI, and low-order modes and highlights the fundamental difference between statistical and physical approaches, with the latter prioritizing the system that generates the activity, with observations and their correlations flowing as corollaries.

Systematic physically based work on EC-FC relationships has rested on spectral analysis in which activity, EC, and FC are all expanded in series of eigenfunctions (also termed natural modes, eigenvectors, eigenmodes, or just modes) of the system, analogous to the natural modes of a violin string. This allows the dominant, large-scale activity and connectivity to be compactly expressed in terms of just a few modes, leading to dramatic simplifications in their representation. For example, the 998 × 998 FC matrix of (Honey et al., 2009) has nearly 500 000 independent elements, but can be reduced to just 998 entries that represent eigenmodes; these in turn are dominated by just a few tens of entries and can often be approximated by ~10 eigenmodes and their amplitudes in applications to EEG, evoked activity and fMRI (Nunez, 1995; Nunez et al., 2001; Robinson et al., 2014, 2016, 2018; Mukta et al., 2019, 2020; Gao and Robinson, 2020). This compact representation is the fundamental reason that fMRI and EEG experiments only robustly detect around 10 resting-state patterns or around 5 microstate patterns, respectively, against a noisy background.

Unlike statistical components extracted by some varieties of independent component analysis (ICA) or principal component analysis (PCA) (Beckmann et al., 2005; Leonardi et al., 2013; Anderson et al., 2014), or clusters of nodes grouped on the basis of similarity of correlation (Yeo et al., 2011), eigenmodes are the natural dynamical modes of the physical system, rather than statistical constructs, and thus reflect connectivity and dynamics directly (Robinson, 2012, 2019; Friston et al., 2014; Robinson et al., 2014, 2016; Gabay et al., 2018). Indeed, if the aim is to probe dynamics of the actual physical brain, rather than treat it as a black-box signal generator, one must go beyond statistics, model-free signal analysis, and phenomenology to dynamics-based methods. In symmetric cases such as those obtained by covariance-based FC, eigenmodes form a complete orthonormal basis, which means that any spatially continuous activity and connectivity whatsoever can be expressed in terms of them (Galán, 2008; Friston et al., 2014; Robinson et al., 2014; Robinson, 2019)—this includes microstates, resting state “networks” (RSNs), and ICA and PCA components (v et al., 1995; Beckmann et al., 2005; Fox and Raichle, 2007; Deco et al., 2011; Anderson et al., 2014)—a key advantage of treating the brain as a physical system.

**Figure 2:**
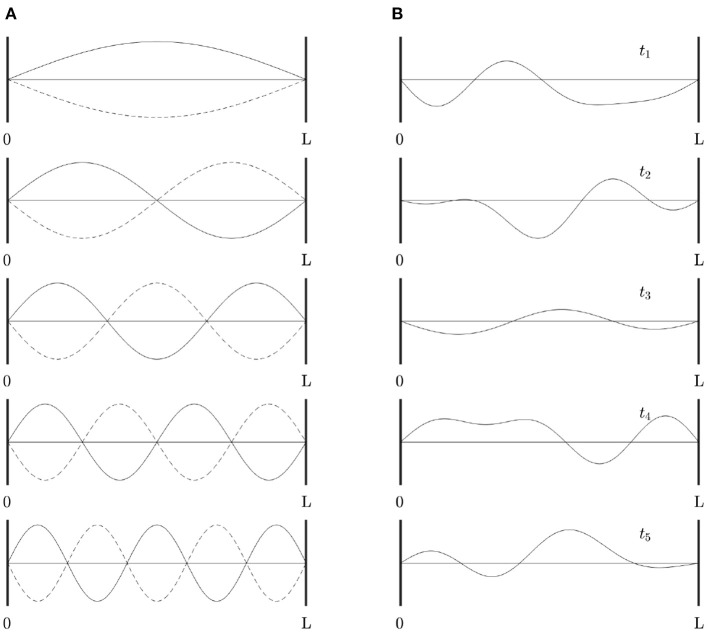
Eigenmodes of a violin string clamped at both ends. **(A)** First five modes, showing waveform at times (solid and dotted) half an oscillation apart. **(B)** Examples of waveforms obtained by superposition of the modes in **(A)** at times *t*_1_ . . . *t*_5_, with frequencies proportional to the mode number as in a real violin string, equal amplitudes, and random initial phases.

For readers who are not familiar with eigenmodes, Figure 2A shows a simple 1D example of the first few eigenmodes of a violin string that is fixed at both ends, where displacement is the analog of changes in brain activity relative to baseline. For a uniform string, each eigenmode is a spatial sinusoid that oscillates in time between the two extremes shown; an integer number of half wavelengths must fit into the length of the string. Key points worth noting are: (i) The eigenmodes are determined by the geometry of the string and its boundary conditions—i.e., its length and fixed endpoints. The endpoints do not move (i.e., no change in activity) but are critical to the properties of the modes. (ii) Each mode occupies the whole string, not a localized region. (iii) Modes overlap in space and every point on the string is part of every mode, although some points in each mode (so-called nodes or zeros) have zero amplitude. (iv) Despite (iii), modes are independent and do not interact, unless nonlinear effects are introduced. Even in the nonlinear case, linear spatial modes remain a useful starting point for spatiotemporal analysis. (v) There is no part of the string that can be excised and said to produce one mode of oscillation. (vi) Each mode, except the lowest, involves both simultaneous positive and negative regions. Hence, anticorrelated regions are just as essential to the dynamics as correlated ones and nodal lines of zero activity change will exist. (vii) Because the lowest mode has the same sign at all points at a given instant, it tends to induce global positive correlations relative to any given point, whereas the contributions from other modes average spatially to zero, so these modes always tend to cause some other points to be anticorrelated with any given point. When this mode is deleted, negative correlations must occur, as has been reported as a result of global signal removal in fMRI (Murphy and Fox, 2017). (viii) Eigenmode expansion is illustrated in Figure 2B, which shows how a complex waveform can be expressed in terms of the amplitudes and phases of the lowest eigenmodes.

The features noted in the previous paragraph are based on methods that have been developed and applied over two centuries in physics and engineering, starting with Fourier (Fourier, 1822; Schiff, 1968; Zwillinger, 1989; Courant and Hilbert, 2004), and whose properties are extremely well understood. These attributes contrast with many inherent in widely used FC analyses (Robinson, 2019): (i) ICA and PCA do not take into account geometry. (ii) Resting state networks are usually constructed to be spatially nonoverlapping. (iii) Use of positive correlations to define “networks” in some analyses neglects dynamically essential negative correlations—the supposedly problematic production of negative correlations when global signals are removed from fMRI (Chen et al., 2011) is actually an automatic consequence of removing activity in the lowest mode, which is the only one with global all-positive correlations. (iv) Thresholding of correlations deletes modal zeros, which have no activity but are critical in determining dynamical properties, and selects a few local regions as being responsible for dynamics. This is akin to retaining just the highest-amplitude regions of the string in Figure 2A, which would certainly not produce a note. (v) The common practice of coarsely discretizing the brain then treating thresholded links between these regions as being an actual brain network with graph-theoretic properties such as degree, clustering, modularity, and hierarchy is a category error (Ryle, 1949) because there is no discrete cortical network with such properties at macroscopic scales. (vi) Many methods ignore the dimensionality and units of the quantities involved, leading to neglect of relative areas of regions of interest, for example.

In this paper we use spectral analysis to quantify dynamic connectivity and constrain its dependence on actual EC changes, transient stimuli, and windowing effects. Specifically, we synthesize multiple results from the literature on eigenmode analysis of brain structure and function to decompose brain activity in terms of long-term average eigenmodes of the covariance of activity. These modes form a complete orthonormal basis, which we demonstrate to be robust to perturbations—so there can be no rapid wholesale changes in these dynamical building blocks. We then use these basis functions to compactly decompose instantaneous brain activity, EC, and FC in terms of mode amplitudes. These are then used to probe short term changes in connectivity, plus the effects of windowing, averaging, natural activity bandwidth, and other phenomena. Similarly, we show how to express evoked responses and the system transfer function in terms of these modes and to approximate them via sparse measurements of evoked responses.

The structure of the paper is as follows: Section 2 briefly overviews the background theory for an interdisciplinary readership. It generalizes key results and demonstrates that eigenmodes of the corticothalamic system have stable spatial structure under moderate perturbations. Section 3 then discusses how to analyze dynamic FC in terms of eigenmodes, examines intrinsic limitations of covariance-based approaches, and points out advantages of a more direct approach via eigenmode coefficients. Examples are provided to illustrate the key results in simple situations, with a summary and discussion in section 5.

## 2. Materials and Methods

In this section we provide the necessary background to our analysis, generalizing and adapting it as required. We first outline neural field theory and its relationship to EC, then introduce eigenmodes and expansions of activity, EC, and FC in terms of them. This summarizes and elucidates prior work, cited below, extends prior results, expresses them in alternative notations that are useful in different contexts, and clarifies several misconceptions. To keep the scope manageable we restrict attention to corticocortical EC and FC but allow for local dynamics, which can include corticothalamic feedbacks (Robinson et al., 2016; Robinson, 2019).

### 2.1. Neural Field Theory

The spatial connectivity scales of interest here range from ~1 mm up to the whole brain, with large-scale patterns seen in microstates and resting state networks having sizes of several cm and up. Connectivity is continuous, not discrete, on the scales of interest (i.e., neurons are not resolved). Hence, we do not need to analyze individual neurons and neural field theory (NFT) is well suited to address activity and connectivity (Nunez, 1995; Deco et al., 2008; Coombes et al., 2014; Robinson, 2019). NFT averages over neural populations to obtain equations for local means of quantities such as afferent activity, soma potential, and firing rate, which underlie observable signals (Nunez, 1995; Jezzard et al., 2001). Some reviews of its many successful applications to predict and analyze experimental results include (Deco et al., 2008; Coombes et al., 2014).

Large scale connectivity is usually assumed to be approximately linear, which is a good approximation because axonal outputs are generally proportional to their inputs even when the points they connect undergo nonlinear dynamics. Hence, we employ the linear limit of NFT here and make as few other assumptions as possible about the specific form of the theory to keep the results as general as possible. We note that if the equations of NFT are discretized (e.g., for numerical analysis or coarse-grained approximation), this yields neural mass theory (NMT), which is often incorrectly asserted to be distinct from NFT. By proceeding from NFT to NMT one obtains correctly weighted connections between discretized spatial neural masses, which is not the case if NMs are simply added to a network such as an observed connectome.

#### 2.1.1. Integral Formulation

**Figure 3:**
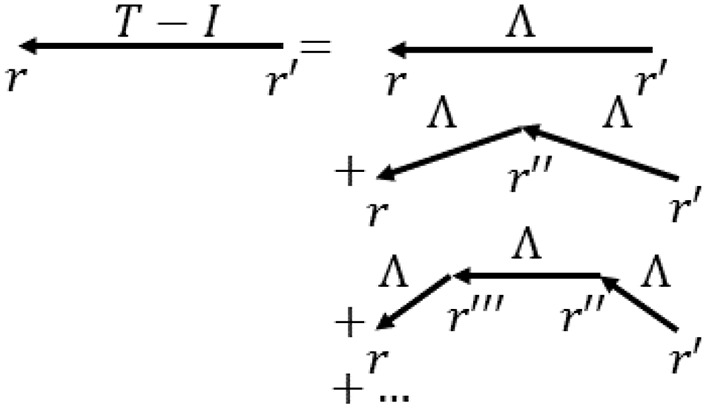
Schematic of the terms in Equation (1) for propagation of activity to *r* = (**r**,*t*) from *r*′ = (**r**′, *t*′). The total propagator *T* is the sum of terms representing direct input *I* plus, on the right, direct propagation Λ, propagation via neural interaction at one intermediate location *r*″, and so forth. Most commonly arrows indicate propagation between cortical locations via white matter fibers, while vertexes denote points where local interactions occur, including via corticothalamic loops.

We assume that experimental signals are linear functions of perturbations *Q*(**r**, *t*) of brain activity from an average steady state. In the linear regime, one has (Robinson, 2019).

(1) $$\begin{array}{l} Q(r,t)=\int \int \Lambda (r,t;r^{\prime },t^{\prime })Q(r^{\prime },t^{\prime })d^{2}r^{\prime }dt^{\prime }+N(r,t), \end{array}$$

where signals travel to a position **r** at time *t* from **r**′ at time *t*′ with a strength Λ, which is the direct effective propagator or direct EC tensor, and *N* represents external input (Robinson, 2012, 2019; Robinson et al., 2014). Figure 3 shows the physical situation corresponding to Equation (1). In Equation (1) local dynamics, including interactions between short-range populations such as interneurons, are absorbed into the structure of Λ (Robinson, 2019), so only long range propagation via white matter fibers is explicit, as in macroscopic observations.

Before proceeding, we note that the linear assumption in Equation (1) is well justified as a starting point: (i) Although the steady states of the brain may be determined nonlinearly, perturbations from them can be linearly approximated. Linear approximations have been successful in describing a wide range of normal phenomena, as noted in the Introduction. Dynamics of phenomena such as seizures require a fully nonlinear approach, but are not considered in the present work. (ii) Correlations of relatively weak activity changes suffice to establish functional connections. (iii) Spatial connectivity is often approximately linear even when local temporal dynamics are nonlinear—output spike rates from each axon closely follow the rate at which spikes are generated at its axonal hillock. (iv) Unless the linear case is understood, it is likely to be premature to try to understand nonlinear aspects of the problem.

One can re-express Equation (1) in the form

(2) $$\begin{array}{l} Q(r,t)=\int \int T(r,t;r^{\prime },t^{\prime })N(r^{\prime },t^{\prime })d^{2}r^{\prime }dt^{\prime }, \end{array}$$

where *T* is the system transfer function, or total EC (Robinson, 2012, 2019). Note that if *N* is a delta function, the response is just *T*, whence we note that *T* is the overall Green function of the system, and corresponds to the widely studied evoked response (Luck and Kappenman, 2012; Robinson, 2012, 2019; Robinson et al., 2018; Henderson et al., 2021).

In a system with static structure (we generalize this in section 3), the correlation function can be used to define a generalized FC via

(3) $$\begin{array}{l} C(r,r^{\prime },\tau )=\int Q(r,t+\tau )Q(r^{\prime },t)dt, \end{array}$$

which is equivalent to selecting the zero-frequency Fourier component in the spectral domain. In practice, one would perform this integral over a window long enough that correlations between activity at opposite ends can be ignored. If the system structure does not depend on time, Λ and *T* depend on time only via the difference τ, so we can use the notations Λ(**r**, **r**′, τ) and *T*(**r**, **r**′, τ). The temporal integrals in Equations (1, 2) then become convolutions and we can Fourier transform vs. time to obtain (Robinson, 2012, 2019).

(4) $$\begin{array}{l} Q(r,\omega )=\int \Lambda (r,r^{\prime },\omega )Q(r^{\prime },\omega )d^{2}r^{\prime }+N(r,\omega ), \end{array}$$

(5) $$\begin{array}{l} =\int T(r,r^{\prime },\omega )N(r^{\prime },\omega )d^{2}r^{\prime }, \end{array}$$

where ω is the angular frequency. Setting τ = 0 in Equation (3) yields the covariance, which is widely used to define functional connectivity in fMRI experiments.

In the commonly considered case of “resting state” activity, in which no task is imposed by an experimenter and the subject is in relaxed surroundings (although the brain is *not* resting but is constantly performing background functions Raichle, 2011), background stimuli span a broad range of spatial and temporal scales after passing through the peripheral nervous system, which also tends to whiten them to make best use of available bandwidth. Consequently, numerous applications to experimental data have shown that background perturbations *N*(**r**, ω) can be approximated by spatially uncorrelated white noise (Robinson et al., 1997, 2002; Deco et al., 2008; van Albada et al., 2010; Abeysuriya et al., 2015), with

(6) $$\begin{array}{l} \int \int N\left(\text{r},t+\tau \right)N^{*}\left({\text{r}}^{\prime },t\right)d^{2}\text{r}dt=N_{0}^{2}\delta^{2}\left(\text{r}-{\text{r}}^{\prime }\right)\delta \left(\tau \right), \end{array}$$

with normalization *N*_0_. We then evaluate the Fourier transform of (3); i.e., the cross-spectral density:

(7) $$\begin{array}{l} C\left(\text{r},{\text{r}}^{\prime },\omega \right)=N_{0}^{2}\int T\left(\text{r},{\text{r}}^{\prime \prime },\omega \right)T^{*}\left({\text{r}}^{\prime },{\text{r}}^{\prime \prime },\omega \right)d^{2}{\text{r}}^{\prime \prime }, \end{array}$$

where the asterisk denotes complex conjugation and the integral is over all **r**″.

A key issue that is largely ignored in the literature is that most of the quantities in the above equations have physical dimensions and units. If these are not included, serious errors can occur (Robinson, 2019), such as equating quantities with different dimensions (e.g., area = volume) or failing to include relative areas when comparing different discretized regions. These dimensions are summarized in Table 1 on the assumption that *Q*(**r**, *t*) has the dimensions of a firing rate.

**Table 1:** Quantities in Equations (1–14) and their SI units.

**Quantity**	**SI Unit**
*Q*(**r**,*t*)	s^−1^
*Q*(**r**,ω)	—
*Q*(ω)	—
Λ(**r**,*t*; **r**′, *t*′)	m^−2^ s^−1^
Λ(**r**,**r**′, ω)	m^−2^
Λ(ω)	m^−2^
$\hat{\Lambda }\left(\omega \right)$	—
*C*(**r**,**r**′, τ)	s^−1^
*C*(**r**,**r**′, ω)	—
Ĉ(ω)	—
*N*_0_	m^2^
*u*_*j*_(**r**)	m^−1^
*u*_*j*_	—
κ_*j*_(*t*)	s^−1^
κ_*j*_(ω)	—

*Not all quantities are listed because N and Q have the same units for the same arguments, as do (i) Λ and T, (ii) u_j_ and v_j_, and (iii) κ_j_, θ_j_, and λ_j_*.

#### 2.1.2. Matrix Approximation

It is often useful to discretize the above equations on a fine enough scale that the discretized quantities faithfully represent the continuous ones. We follow this approach as being the most convenient to present a number of key results used below; but revert to the continuous representation when required. Most importantly, we do not make the mistake of viewing this discretization as corresponding to an actual discrete macroscopic cortical network that can be analyzed via graph theory—no such network exists. Data are always recorded in discretized form, but not necessarily resolved finely enough to represent the underlying dynamics faithfully.

For now we limit attention to Equations (4–8) and define a discrete set of positions **r**^*j*^ which are viewed as elements of a column vector *r* (we use superscripts to denote vector and matrix elements to avoid confusion with subscripts, which are used below to denote different eigenvectors and their amplitudes). The corresponding *Q*(**r**, ω) and *N*(**r**, ω) are also column vectors so we write Equation (4) as (Robinson, 2019).

(8) $$\begin{array}{l} Q\left(\omega \right)\approx \hat{\Lambda }\left(\omega \right)Q\left(\omega \right)+N\left(\omega \right), \end{array}$$

where the elements of the square matrix $\hat{\Lambda }$ are

(9) $$\begin{array}{l} {\hat{\Lambda }}^{jk}\left(\omega \right)=\Lambda^{jk}\Delta S^{k}, \end{array}$$

Δ*S*^*k*^ being the discrete piece of cortical surface area represented by *r*^*k*^. The approximation (9) becomes more accurate as the discretization becomes finer; an analogous definition is used for $\hat{T}$. Strictly, $\hat{\Lambda }$ is a tensor because its rows and columns are indexed by 2D position, but this does not affect the analysis below. In a similar way, if we divide by the normalization $N_{0}^{2}$ of the assumed white noise to obtain a normalized version Ĉ of *C*, Equations (5, 7) become

(10) $$\begin{array}{l} Q\left(\omega \right)=\hat{T}\left(\omega \right)N\left(\omega \right), \end{array}$$

(11) $$\begin{array}{l} Ĉ\left(\omega \right)=\hat{T}\left(\omega \right){\hat{T}}^{†}\left(\omega \right), \end{array}$$

where the dagger indicates the Hermitian conjugate (Galán, 2008; Pernice et al., 2011; Robinson, 2012; Pinotsis et al., 2013; Friston et al., 2014; Robinson et al., 2014). The covariance, which is the most commonly used form of FC is given by Equation (3) with τ = 0, which is proportional to *C*(**r**, **r**′, ω = 0). Most commonly, the time average is subtracted from the signals before the covariance is computed and sometimes the result is normalized.

Using the above notation, Equations (8, 10) yield (Galán, 2008; Robinson et al., 2014, 2016; Robinson, 2019).

(12) $$\begin{array}{l} \hat{T}\left(\omega \right)={\left[I-\hat{\Lambda }\left(\omega \right)\right]}^{-1}, \end{array}$$

(13) $$\begin{array}{l} =I+\hat{\Lambda }\left(\omega \right)+{\hat{\Lambda }}^{2}\left(\omega \right)+\ldots , \end{array}$$

(14) $$\begin{array}{l} \hat{\Lambda }=I-{\hat{T}}^{-1}, \end{array}$$

where *I* is the unit matrix, the superscript −1 indicates the matrix inverse, and (13) applies within the radius of convergence. The coordinate equivalent of the formal expansion in Equation (13) is Equation (14) of Robinson (2019). In Equation (12), $\hat{T}$ is the total response, whereas $\hat{\Lambda }$ is the part of the response that travels directly from source to destination, ${\hat{\Lambda }}^{2}$ has one intermediate interaction, and so forth (Galán, 2008; Adachi et al., 2012; Robinson, 2012, 2019; Mehta-Pandejee et al., 2017; Tewarie et al., 2020); $\hat{T}$ and $\hat{\Lambda }$ are thus total and direct ECs.

#### 2.1.3. Perturbation Analysis

As noted above EC can include transient modifications that are due to gain changes that can be approximated as part of the linear response to stimuli (Koch, 1999; Rennie et al., 1999, 2002; Robinson and Roy, 2015; Babaie-Janvier and Robinson, 2020). We treat such effects via perturbation analysis and, in this subsection only, superscript quantities (0) for unperturbed values and (1) to denote perturbations. In this case, we can generalize Equation (8) to yield the following relationship:

(15) $$\begin{array}{l} Q^{\left(1\right)}\left(\omega \right)={\hat{\Lambda }}^{\left(0\right)}\left(\omega \right)Q^{\left(1\right)}\left(\omega \right)+{\hat{\Lambda }}^{\left(1\right)}\left(\omega \right)Q^{\left(0\right)}\left(\omega \right)+N^{\left(1\right)}\left(\omega \right). \end{array}$$

This can be understood if we note that Equation (1) is written on the assumption of static $\hat{\Lambda }=\Lambda^{\left(0\right)}$, so perturbations *Q*^(1)^ arise from the effect of ${\hat{\Lambda }}^{\left(0\right)}$ on perturbations *Q*^(1)^ and direct drive by the inputs *N*^(1)^. However, if $\hat{\Lambda }$ is perturbed by an amount ${\hat{\Lambda }}^{\left(1\right)}$, there will be another contribution to *Q*^(1)^ from the effect of the perturbation on the steady state activity *Q*^(0)^.

We now suppose that $\hat{\Lambda }$ is affected by spatially local feedbacks due to habituation, facilitation, and other processes. In coordinate notation, we assume that changes in Λ^(1)^(**r**, **r**′, τ) are driven by local activity arriving at **r** from **r**′ and obey a temporal differential equation. Then, writing indexes explicitly, one has

(16) $$\begin{array}{l} {\left[{\hat{\Lambda }}^{\left(1\right)}(\omega )\right]}^{ij}=Z(\omega )\{{\left[{\hat{\Lambda }}^{\left(0\right)}(\omega )\right]}^{ij}{\left[Q^{\left(1\right)}(\omega )\right]}^{j}\;\\ \;+{\left[{\hat{\Lambda }}^{\left(1\right)}(\omega )\right]}^{ij}{\left[Q^{\left(0\right)}(\omega )\right]}^{j}\}, \end{array}$$

where Z(ω) is the relevant differential operator, which is assumed to be the same at all points, but this assumption can be relaxed. The other factor on the right is the activity arriving at **r** from **r**′, labeled *i* and *j*, respectively. Equation (16) can be solved for ${\left[{\hat{\Lambda }}^{\left(1\right)}\right]}^{ij}$, whence (15) can be written as in Equation (8), but with

(17) $$\begin{array}{l} \hat{\Lambda }={\hat{\Lambda }}^{\left(0\right)}+\hat{L}, \end{array}$$

(18) $$\begin{array}{l} {\left[\hat{L}\right]}^{ij}=\frac{Z\left(\omega \right){\left[{\hat{\Lambda }}^{\left(0\right)}\right]}^{ij}{\left[Q^{\left(0\right)}\right]}^{j}}{1-Z\left(\omega \right){\left[Q^{\left(0\right)}\right]}^{j}}, \end{array}$$

and the transfer function is modified to

(19) $$\begin{array}{l} \hat{T}={\left[I-{\hat{\Lambda }}^{\left(0\right)}-\hat{L}\right]}^{-1}. \end{array}$$

**Figure 4:**
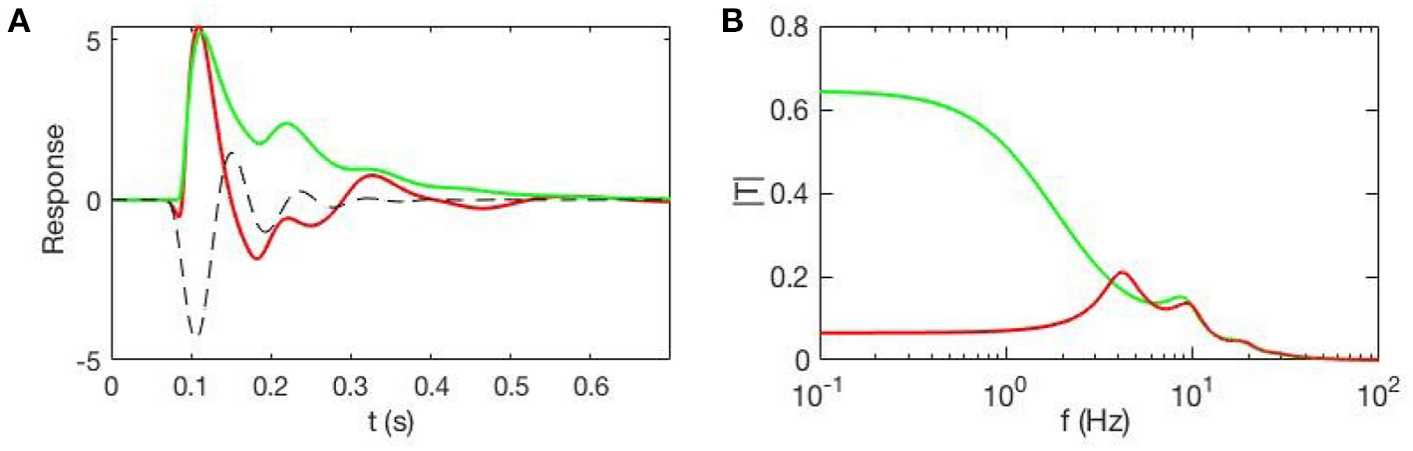
Evoked responses and transfer functions for cases with static EC (green) and dynamic EC (red) starting from the same initial state. **(A)** Model cortical evoked responses. The dashed black line shows the contribution of the EC dynamics and adds to the green curve to give the red one. **(B)** Magnitudes of the corresponding transfer functions.

Figure 4 shows an example of cortical evoked responses and transfer functions from inputs to cortical activity, with and without accompanying EC changes due to gain evolution. These curves are obtained from a full analysis whose details can be found in Babaie-Janvier and Robinson (2020), showing that evoked EC changes (and, by implication, evoked FC changes) can be substantial. This result implies that one cannot separate the contributions from ${\hat{\Lambda }}^{\left(0\right)}$ and $\hat{L}$ without a model that allows the form and dynamics of Z to be estimated.

Equations (11) and (12) explicitly show the connections between direct EC, total EC, and resting state FC, thereby obviating much of the huge literature devoted to probing these connections via statistical methods such as correlations between connectivity patterns or connection matrix entries (Greicius et al., 2009; Sporns, 2011; Messe et al., 2014, 2015; Fornito et al., 2016; Preti et al., 2017). Equations (15–19) show that the connections between structural connectivity (SC; i.e., anatomy) and EC are more complex because it is not only the presence of connections that is relevant, but also their time-dependent strength (Britz et al., 2010; Bassett et al., 2011; Deco and Jirsa, 2012; Kopell et al., 2014; Hansen et al., 2015; Leonardi et al., 2015; Deco et al., 2017; Babaie-Janvier and Robinson, 2019, 2020). However, because only the EC is directly relevant to the dynamics and the FC, the SC-EC relationship can be viewed as a separate issue.

### 2.2. Eigenmode Expansion: Spectral Analysis

The matrix Ĉ in Equation (11) is Hermitian, so its spatial eigenfunctions *u*_*j*_ form an orthonormal basis set (Schiff, 1968; Courant and Hilbert, 2004), and are written as column vectors that approximate the continuum eigenmodes and satisfy

(20) $$\begin{array}{l} Ĉ\left(\omega \right)u_{j}=\kappa_{j}\left(\omega \right)u_{j}, \end{array}$$

for some eigenvalues κ_*j*_(ω), which are discussed below. Note that in a static structure the *u*_*j*_ do not depend on time, just as for the shapes of the sine waves in Figure 2.

Analogous equations to (20) apply for $\hat{\Lambda }$ and $\hat{T}$. Given Equations (12, 13) these quantities commute and thus have the same eigenvectors, *v*_*j*_, with

(21) $$\begin{array}{l} \hat{\Lambda }\left(\omega \right)v_{j}=\lambda_{j}\left(\omega \right)v_{j}, \end{array}$$

(22) $$\begin{array}{l} \hat{T}\left(\omega \right)v_{j}=\theta_{j}\left(\omega \right)v_{j}. \end{array}$$

If the connectivities are symmetric [i.e., Λ(**r**, *t*, **r**′, *t*′) = Λ(**r**′, *t*, **r**, *t*′)] then *v*_*j*_ = *u*_*j*_, so the eigenmodes of the white-noise-driven covariance are the same as those of the transfer function and the activity. We assume symmetry for the remainder of the present work.

Because the eigenfunctions *u*_*j*_ form a complete orthonormal set, one can expand arbitrary activity in terms of them (Schiff, 1968; Courant and Hilbert, 2004); i.e.,

(23) $$\begin{array}{l} Q\left(\omega \right)=\underset{j}{\sum }c_{j}\left(\omega \right)u_{j}, \end{array}$$

(24) $$\begin{array}{l} c_{j}\left(\omega \right)=Q^{T}\left(\omega \right)u_{j}, \end{array}$$

(25) $$\begin{array}{l} u_{j}^{T}u_{k}=\delta_{jk}, \end{array}$$

where δ_*jk*_ is the Kronecker delta. Alternatively, in coordinate notation, one has

(26) $$\begin{array}{l} Q\left(\text{r},t\right)=\underset{j}{\sum }c_{j}\left(t\right)u_{j}\left(\text{r}\right), \end{array}$$

(27) $$\begin{array}{l} c_{j}\left(t\right)=\int u_{j}^{*}\left(\text{r}\right)Q\left(\text{r},t\right)d^{2}\text{r}, \end{array}$$

(28) $$\begin{array}{l} \delta_{jk}=\int u_{j}^{*}\left(\text{r}\right)u_{k}\left(\text{r}\right)d^{2}\text{r}. \end{array}$$

One can also expand the connectivities in terms of eigenfunctions via spectral decomposition. In the symmetric case, one has

(29) $$\begin{array}{l} \hat{\Lambda }\left(\omega \right)=UL\left(\omega \right)U^{†}, \end{array}$$

(30) $$\begin{array}{l} \hat{T}\left(\omega \right)=U\Theta \left(\omega \right)U^{†}, \end{array}$$

(31) $$\begin{array}{l} Ĉ\left(\omega \right)=UK\left(\omega \right)U^{†}, \end{array}$$

where *U* is the unitary matrix whose columns are the orthonormal eigenvectors and *L*, Θ, and *K* are diagonal matrices whose nonzero entries are the corresponding eigenvalues Robinson et al. (2014); Robinson (2019), which satisfy

(32) $$\begin{array}{l} \kappa_{j}\left(\omega \right)=|\theta_{j}\left(\omega \right){|}^{2}, \end{array}$$

(33) $$\begin{array}{l} \theta_{j}\left(\omega \right)={\left[1-\lambda_{j}\left(\omega \right)\right]}^{-1}, \end{array}$$

using Equations (11, 12), respectively. From Equations (10, 30) we have

(34) $$\begin{array}{l} N={\hat{T}}^{-1}Q=U\Theta^{-1}U^{†}Q, \end{array}$$

which describes how to infer the input from measurements of the activity using *T*. Terms in the power series in Equation (13) have the form [Λ(ω)]^*m*^ = *U*[*L*(ω)]^*m*^*U*^†^.

The coordinate space equivalents of Equations (29–31) are

(35) $$\begin{array}{l} \Lambda (r,r^{\prime },\tau )=\sum_{j}u_{j}^{*}(r)u_{j}(r^{\prime })\lambda_{j}(\tau ), \end{array}$$

(36) $$\begin{array}{l} T(r,r^{\prime },\tau )=\sum_{j}u_{j}^{*}(r)u_{j}(r^{\prime })\theta_{j}(\tau ), \end{array}$$

(37) $$\begin{array}{l} C(r,r^{\prime },\tau )=\sum_{j}u_{j}^{*}(r)u_{j}(r^{\prime })\kappa_{j}(\tau ), \end{array}$$

where τ = *t* − *t*′. Each eigenvector *u*_*j*_, or *u*_*j*_(**r**) in coordinate notation, extends over the whole system, with every point being part of every eigenfunction, as in the example in Figure 2. However, the diagonal representations in Equations (29–31) mean that these discrete modes are not coupled, are completely independent in the linear regime, and can only be linearly excited by input that has a component in the same mode. Eigenmodes thus form a fundamentally discrete set of dynamical building blocks, unlike statistically derived and/or phenomenological resting state patterns, for example.

Yeo et al. (2011) and others have clustered discretized brain regions based on their overall patterns of functional connectivity defined by the covariance. Their analyses also employed thresholding and sparsification of connection matrices, which we do not do here. Rather, we show that their measure of similarity of connectivity can be expressed in simple form using the present methods. The similarity *S*(**r**_1_, **r**_2_) of FC patterns emanating from **r**_1_ and **r**_2_ is the dot product between the relevant vectors of functional connections:

(38) $$\begin{array}{l} S\left({\text{r}}_{1},{\text{r}}_{2}\right)=\int C\left({\text{r}}_{1},{\text{r}}^{\prime }\right)C\left({\text{r}}^{\prime },{\text{r}}_{2}\right)d{\text{r}}^{\prime }, \end{array}$$

where we note that the covariance is symmetric and omit the argument ω = 0. In matrix notation, the integral corresponds to multiplication, so

(39) $$\begin{array}{l} Ŝ={Ĉ}^{2}, \end{array}$$

and the eigenvectors of Ŝ are those of Ĉ, while its *j*th eigenvalue is just $\kappa_{j}^{2}$.

**Figure 5:**
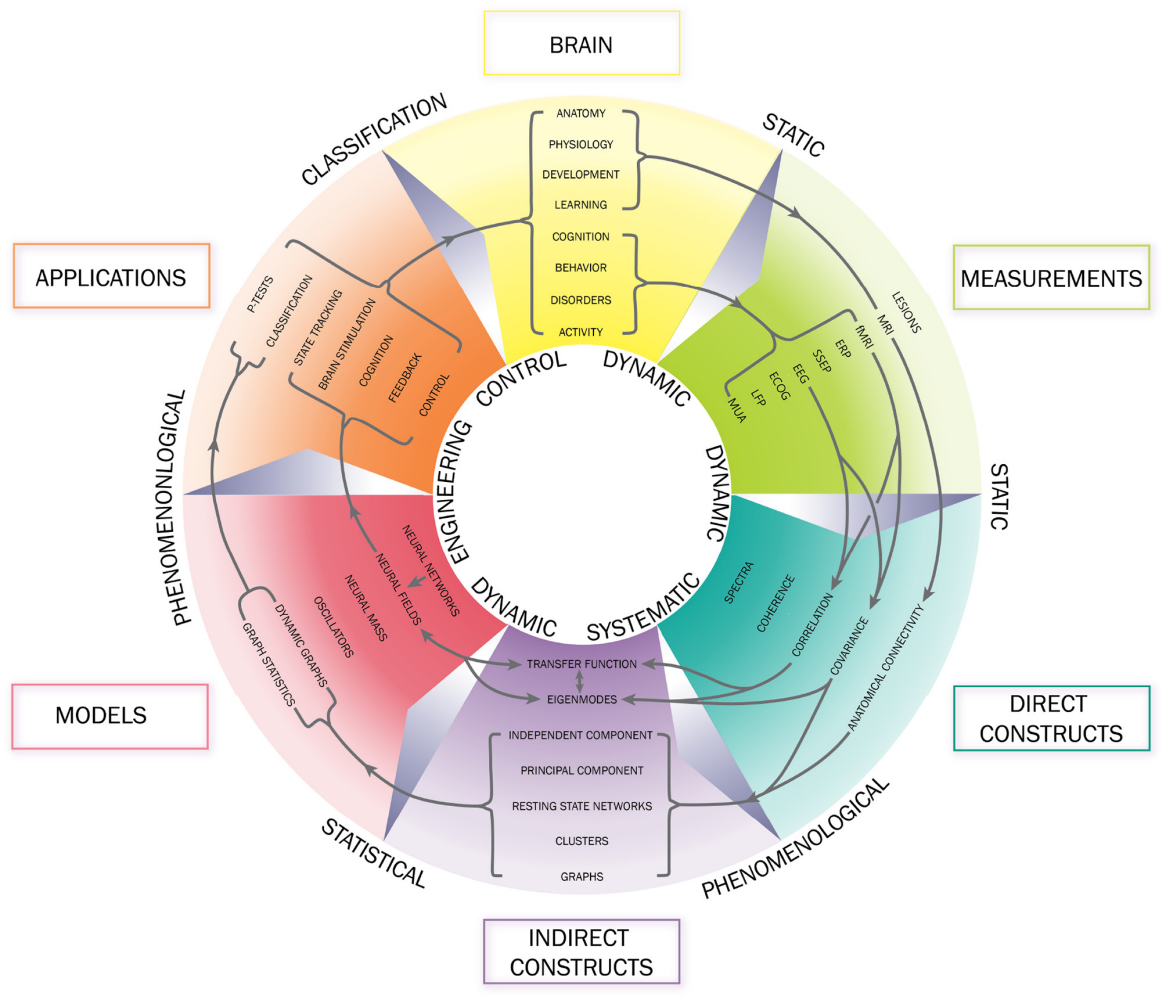
Schematic of some of the relationships of brain data, analyses, and applications. Clockwise from the top, categories of brain observables are shown, followed by measurements, direct and indirect data analyses, resulting models, and applications. In general, smaller radii refer to more dynamic and physical aspects, while larger radii relate to more static and phenomenological aspects. (Of course, some physical aspects are static and some dynamical analyses are phenomenological.) Arrows show a subset of the relationships used in the field.

The arrows in the schematic Figure 5 show how the various quantities above, and many others used in the field, are related. Note that phenomenological statistical approaches are located toward the circumference while more detailed physical ones are toward the center.

### 2.3. Spatiotemporal Structure

So far, we have not introduced any particular theory of the dynamics of brain activity. To predict the spatial structure of modes, one needs to introduce a specific model and geometry; although modes can be inferred from FC data without doing this, via Equations (29–33) (Robinson et al., 2014; Robinson, 2019). We begin with the illustrative case of a violin string from Figure 2, then proceed to a spherical-cortex case, which forms the basis for many of the subsequent arguments where we generalize to a convoluted cortex. The methods used are standard approaches in the physics and engineering literature, but have not yet been widely used in connectomics; more details can be found in standard references such as Schiff (1968), Courant and Hilbert (2004), and Ogata and Yang (1970).

#### 2.3.1. Violin String Analog System

The equation of motion of an undamped, undriven violin string in response to an impulse stimulus at *x* = 0 and *t* = 0 is

(40) $$\begin{array}{l} \frac{\partial^{2}Q\left(x,t\right)}{\partial t^{2}}-v^{2}\frac{\partial^{2}Q\left(x,t\right)}{\partial x^{2}}=\delta \left(x\right)\delta \left(t\right), \end{array}$$

where *x* is the position on the string, *v* is the velocity of waves, and *Q* here represents the displacement perpendicular to the string, which is the analog of brain activity.

The transfer function is the response to an impulse stimulus (Zwillinger, 1989; Courant and Hilbert, 2004). Hence *T* is obtained by Fourier transforming Equation (40) vs. position and time to give

(41) $$\begin{array}{l} T\left(k,\omega \right)\propto \frac{1}{k^{2}v^{2}-\omega^{2}}, \end{array}$$

where *k* is the wave number, which is the spatial analog of ω.

Because the properties of the violin string do not change in time, we can solve Equation (40) by the method of separation of variables, in which we make the ansatz that *Q*(*x, t*) is a sum of solutions of the form

(42) $$\begin{array}{l} Q\left(x,t\right)=u\left(x\right)\theta \left(t\right). \end{array}$$

By substituting the form (42) into Equation (40) and dividing by *Q*(*x, t*), we find

(43) $$\begin{array}{l} \frac{1}{v^{2}\theta \left(t\right)}\frac{\partial^{2}\theta \left(t\right)}{\partial t^{2}}=\frac{1}{u\left(x\right)}\frac{\partial^{2}u\left(x\right)}{\partial x^{2}}=-k^{2}, \end{array}$$

where *k* is a separation constant that arises because the other two members of Equation (43) are independent of *x* and *t*, respectively, so they must both equal a common constant.

Taking the spatial part of Equation (43) yields the Helmholtz equation and we find

(44) $$\begin{array}{l} u\left(x\right)=a\cos\left(kx\right)+b\sin\left(kx\right), \end{array}$$

for some constants *a* and *b*. The only orthonormal solutions that are zero at *x* = 0, *L* are

(45) $$\begin{array}{l} u_{j}\left(x\right)={\left(2/L\right)}^{1/2}\sin\left(k_{j}x\right), \end{array}$$

where *L* is the length of the string. The permitted solutions correspond to the eigenvalues

(46) $$\begin{array}{l} k_{j}=j\pi /L, \end{array}$$

where *j* = 1, 2, 3, ….

Once the eigenvalues have been determined, they can be substituted into the temporal part of Equation (43), the dispersion equation, to yield

(47) $$\begin{array}{l} \theta_{j}\left(t\right)=\sin\left(\omega_{j}t+\psi_{j}\right), \end{array}$$

(48) $$\begin{array}{l} \omega_{j}=k_{j}v, \end{array}$$

where ψ_*j*_ is the phase at *t* = 0. Hence, the displacement corresponding to a given eigenfunction has a profile that oscillates sinusoidally in time as well as space. The coefficients of the modes can be determined from Equation (27) at *t* = 0. The standing waves given by Equations (42, 44, 47) can equally well be interpreted as superpositions of equal-amplitude left- and right-propagating traveling waves.

This simple example epitomizes the fact that boundary conditions—where the displacement is zero in this case—break the symmetry to select a discrete set of eigenfunctions with specific spatial structure. All modes except the lowest have both positive and negative regions, so methods that seek patterns on the basis of clustering around points of high positive correlation ignore fundamental features of the actual brain dynamics, just as a children's seesaw without both its “negatively correlated” ends cannot function. It is also worth stressing that the 1D Laplacian operator ∂^2^/∂*x*^2^ appears in Equation (40), which is a wave equation. A common error in the literature is to presume that the appearance of this operator means that the dynamics is diffusive Atasoy et al. (2016, 2018); although the spatial eigenfunctions are the same in both cases, the dynamics are very different and must not be confused. Indeed the diffusion equation and the wave equation are in different families of partial differential equations—parabolic and hyperbolic, respectively (Zwillinger, 1989; Courant and Hilbert, 2004). Spatial spreading of activity via wave propagation can also be treated by matrix methods that include all possible multistep paths with appropriate time delays (Galán, 2008; Robinson et al., 2016; Mehta-Pandejee et al., 2017; Robinson, 2019; Tewarie et al., 2020).

#### 2.3.2. Spherical Case

Many common neural field brain models have a transfer function of the form

(49) $$\begin{array}{l} T\left(\text{k},\omega \right)=\frac{A\left(\omega \right)}{k^{2}v^{2}+q^{2}\left(\omega \right)v^{2}}, \end{array}$$

which generalizes Equation (41) to embody an overall frequency envelope *A*(ω) plus Laplacian spatial coupling and complicated local dynamics described by *q*(ω), which can involve multiple populations of cortical and thalamic neurons (Nunez, 1995; Robinson et al., 2002; Deco et al., 2008; Robinson, 2019); the details of *A*(ω) and *q*(ω) do not concern us here but can be found in the references cited. It is worth noting that the dynamics described by this type of transfer function have been widely verified against experiment (see Jirsa and Haken, 1996; Robinson et al., 1997, 2002, 2014, 2016; Deco et al., 2008 and the references cited therein) and the spatial coupling implied by the Laplacian operator closely matches that seen in the brain (Nunez, 1995; Robinson et al., 1997, 2016; Nunez et al., 2001; Mukta et al., 2019, 2020) and yields eigenfunctions that are very similar to those obtained from connectivity matrices and anatomical studies (Robinson et al., 1997, 2016; Braitenberg and Schüz, 1998). Moreover, the topology of a sphere is the same as that of a brain hemisphere, aside from the 0.5% lacuna where the corpus callosum passes, so it forms a useful starting point for mapping and perturbation analysis (Jirsa et al., 2001; Robinson et al., 2016; Gabay et al., 2018).

To obtain spatial eigenmodes on a sphere of radius *R*_*s*_ we introduce the usual spherical coordinates ϑ and φ and separate variables in these coordinates and time. This yields discrete spatial eigenmodes *u*_*j*_ on the sphere. In this case, the *u*_*j*_ are termed spherical harmonics and written in the notation *Y*_*lm*_, with

(50) $$\begin{array}{l} Y_{lm}\left(\vartheta ,\varphi \right)=c_{lm}P_{l}^{\left|m\right|}\left(\cos\vartheta \right)e^{im\varphi }, \end{array}$$

(51) $$\begin{array}{l} c_{lm}={\left[\frac{\left(l-|m|\right)!\left(2l+1\right)}{4\pi \left(l+|m|\right)!}\right]}^{1/2}, \end{array}$$

where *l* = 0, 1, 2, … and *m* = −*l*, −*l* + 1, …, *l*, and $P_{l}^{\left|m\right|}$ is an associated Legendre function (Dunster, 2010). Real-valued eigenmodes $Y_{l}^{m}$ can be constructed as linear combinations of *Y*_*lm*_ and *Y*_*l*, −*m*_ that give sine and cosine dependences on φ (Dunster, 2010); viz,

(52) $$\begin{array}{l} Y_{l}^{m}={\left(-1\right)}^{m}\left(Y_{lm}+Y_{lm}^{*}\right)/\sqrt{2},\;m>0;\\ \;=Y_{lm},\;m=0;\\ \;=-i{\left(-1\right)}^{m}\left(Y_{l|m|}-Y_{l|m|}^{*}\right)/\sqrt{2},\;m<0. \end{array}$$

**Figure 6:**
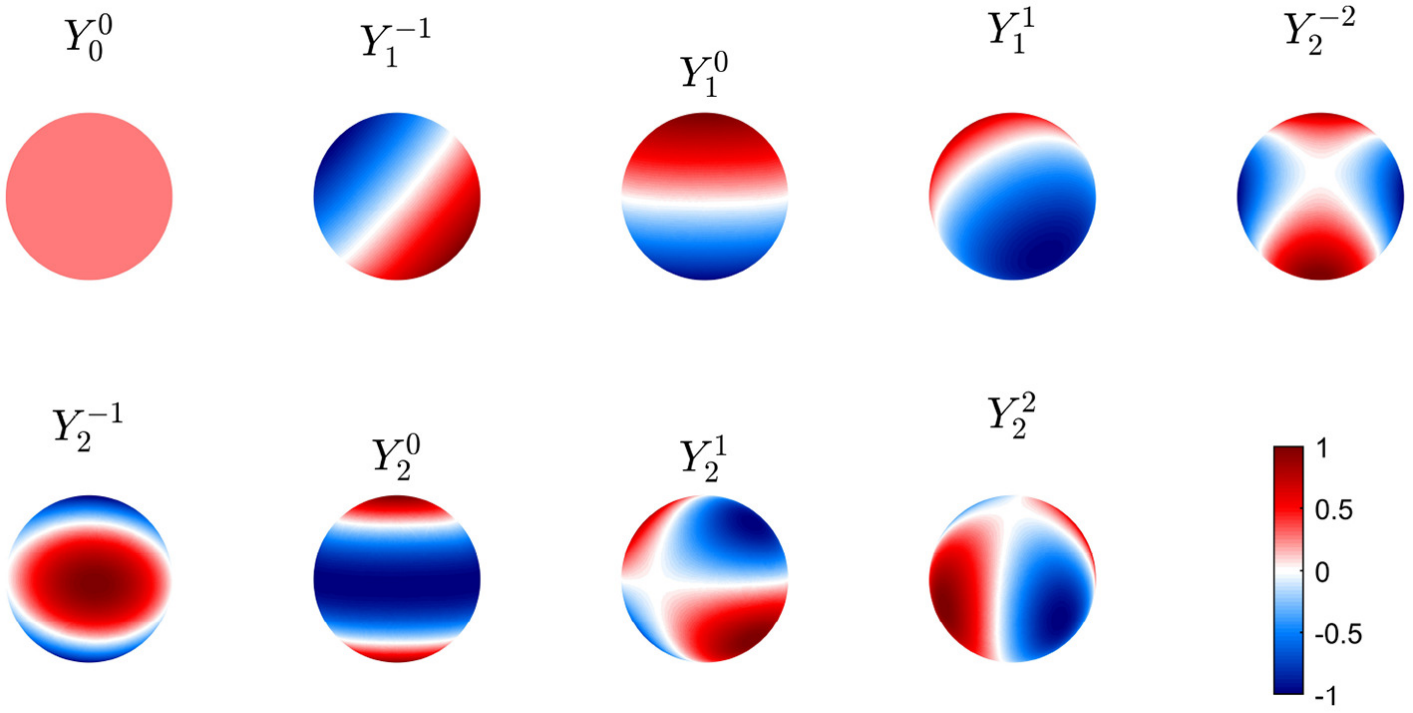
First nine real eigenmodes $Y_{l}^{m}$ on a spherical cortex. In each case the mode is normalized to a maximum amplitude of unity and regions of opposite sign are shown red and blue as in the color bar.

The first nine of these modes are shown in Figure 6 in real form, which is more convenient for plotting and interpretation, although the complex form in Equation (50) is easier for the analytic work below. Note that symmetry implies that these modes can be rotated through any angle on the sphere and still remain eigenmodes; however, nonuniformity breaks this symmetry and removes this degeneracy.

The corresponding eigenvalues are

(53) $$\begin{array}{l} k_{lm}^{2}=l\left(l+1\right)/R_{s}^{2}, \end{array}$$

which are independent of *m* because of spherical symmetry. Setting the denominator of *T*(**k**, ω) to zero implies that the wave dispersion equation is

(54) $$\begin{array}{l} k_{lm}^{2}+q^{2}\left(\omega \right)=0. \end{array}$$

Unlike Equation (49), when Equation (54) is solved for ω it does not usually give a unique solution for a given *k*_*lm*_ because of the complex dynamics it embodies; rather, for each mode it typically gives rise to a series of frequency resonances, to which we return below (Robinson et al., 2002; Robinson, 2003; Babaie-Janvier and Robinson, 2018, 2019; Gabay et al., 2018). Oscillations at the resonant frequency cause the modes to change back and forth in sign as time passes.

Although the functional form of the spatial eigenfunctions is more complex on a sphere than a violin string, the same key aspects remain: they are discrete with eigenvalues determined by the boundary conditions (a spherical topology and geometry here), overlap in space because each extends over the entire cortex, and have essential anticorrelated regions.

#### 2.3.3. Convoluted Cortex

**Figure 7:**
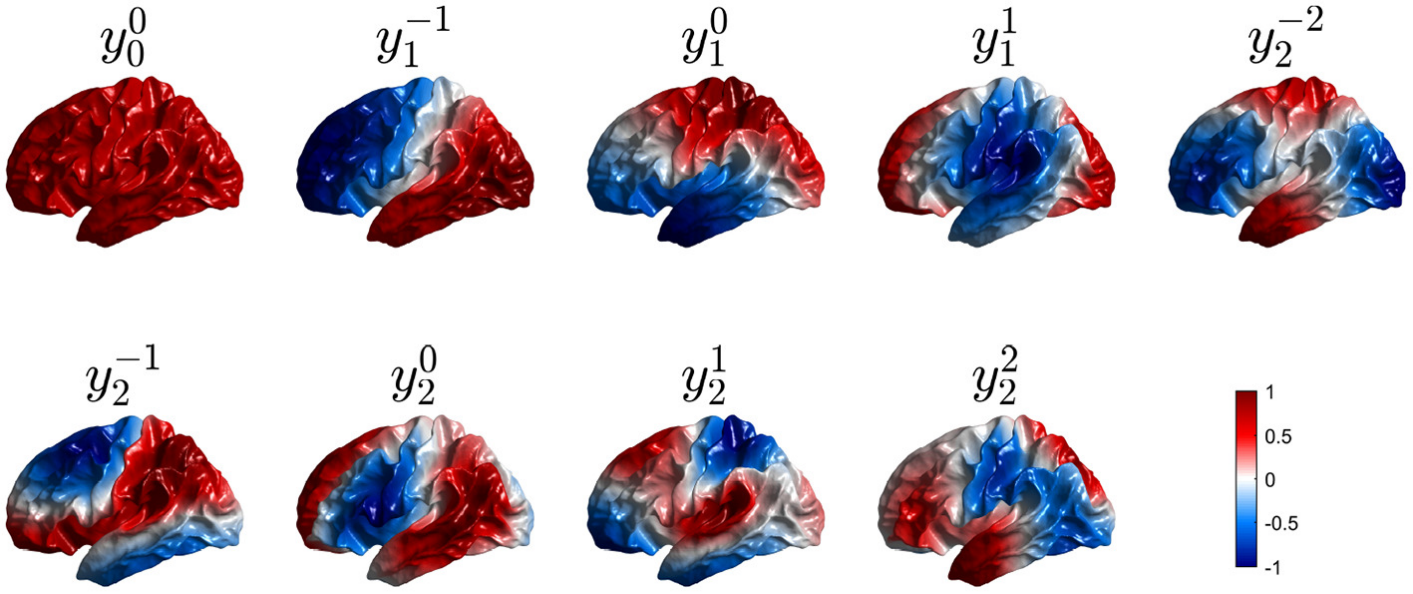
First nine real eigenmodes on a convoluted cortical hemisphere. In each case they are labeled $y_{l}^{m}$ by analogy with the $Y_{l}^{m}$ and normalized to a maximum value of unity; regions of opposite polarity are colored red and blue as shown in the color bar.

Solution of the Helmholtz equation on the highly convoluted cortex cannot be done in closed analytical form because of the complex geometry, although the topology of a cortical hemisphere still remains that of a sphere if one neglects small gaps such as where the corpus callosum passes through, as we justify below. Numerical solution of the NFT equations yields the real modes $y_{l}^{m}$ shown in Figure 7. An appropriate choice of coordinates yields $y_{l}^{m}=Y_{l}^{m}$ in the spherical limit, although more generally, the perturbed eigenfunction $y_{l}^{m}$ is a more general linear combination of the $Y_{l^{\prime }}^{m^{\prime }}$ dominated by *l*′ = *l* (Robinson et al., 2016). Alternatively, solution of the connection matrix $\hat{\Lambda }$ obtained from diffusion imaging yields almost identical spatial structure (Robinson et al., 2016). Unlike the spherical case, the eigenvalues *k*_*lm*_ are not degenerate because spherical symmetry has been broken by the convolutions, which affect the Laplacian (Jirsa et al., 2001; Robinson et al., 2016; Gabay and Robinson, 2017).

Much insight into the symmetry breaking and resulting effects on eigenvalues has been obtained by treating the effects of the convolutions on the Laplacian as perturbations of the spherical case and applying standard perturbation theory from quantum physics to calculate the convoluted eigenmodes as perturbed versions of the spherical ones, with the perturbations expressed as sums over the unperturbed modes (Schiff, 1968; Gabay and Robinson, 2017). This proceeds by using the standard Freesurfer mapping (Fischl et al., 1999; Robinson et al., 2016; Gabay and Robinson, 2017) to map the convoluted surface to the sphere (and vice versa) so as to assign spherical coordinates to every point for the purposes of calculation. The Laplacian on the convoluted surface is likewise mapped to the sphere and the difference from the corresponding spherical value is used as a perturbation.

**Figure 8:**
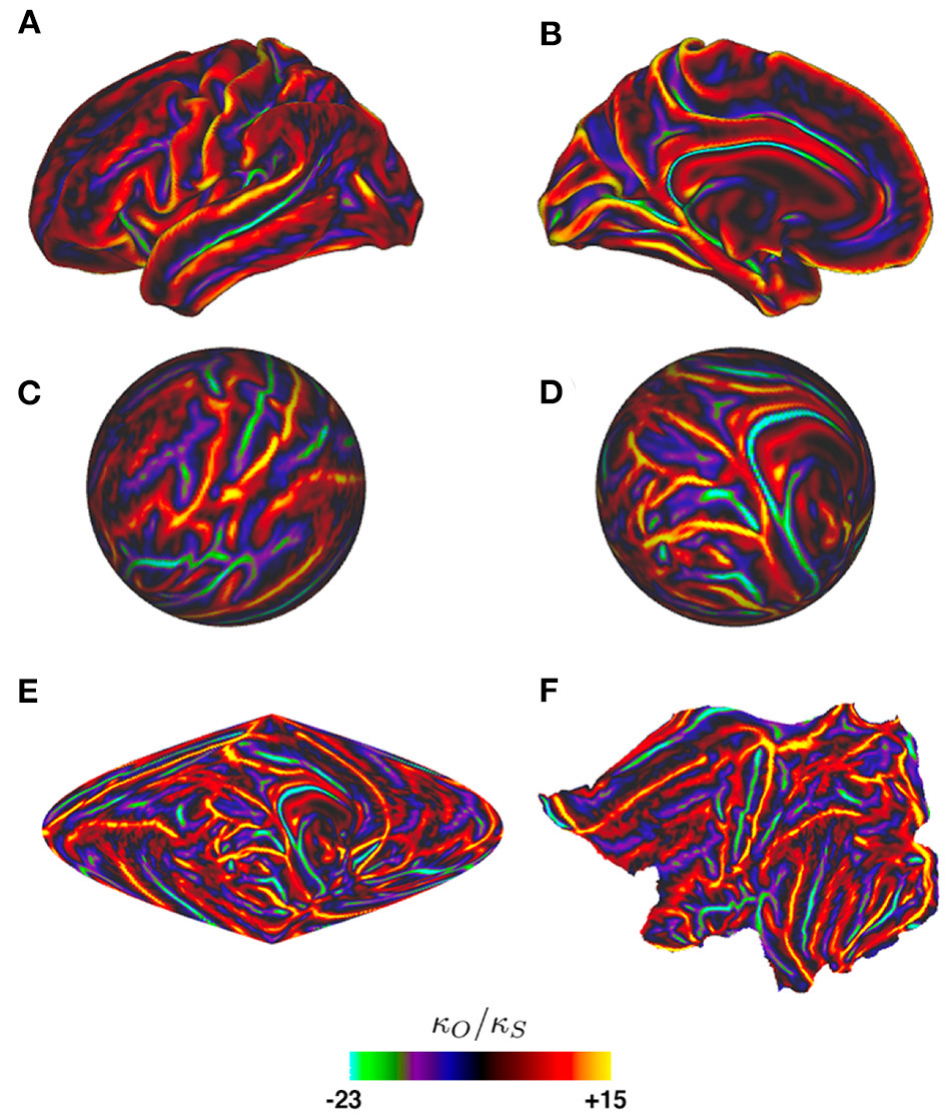
Ratio κ_*O*_/κ_*s*_ of one cortical hemisphere's local curvature to that of an equal-area sphere vs. position, as indicated by the color bar. Surfaces are of an average cortical template from the Human Connectome Project (Glasser et al., 2016). **(A)** Lateral view of the hemisphere. **(B)** Medial view. **(C)** Spherical projection corresponding to **(A)**, mapped to spherical coordinates via the standard Freesurfer mapping (Fischl et al., 1999). **(D)** Spherical projection corresponding to **(B)**. **(E)** Sinusoidal projection of the whole hemisphere. **(F)** Flat projection of the hemisphere (Fischl et al., 1999).

**Figure 9:**
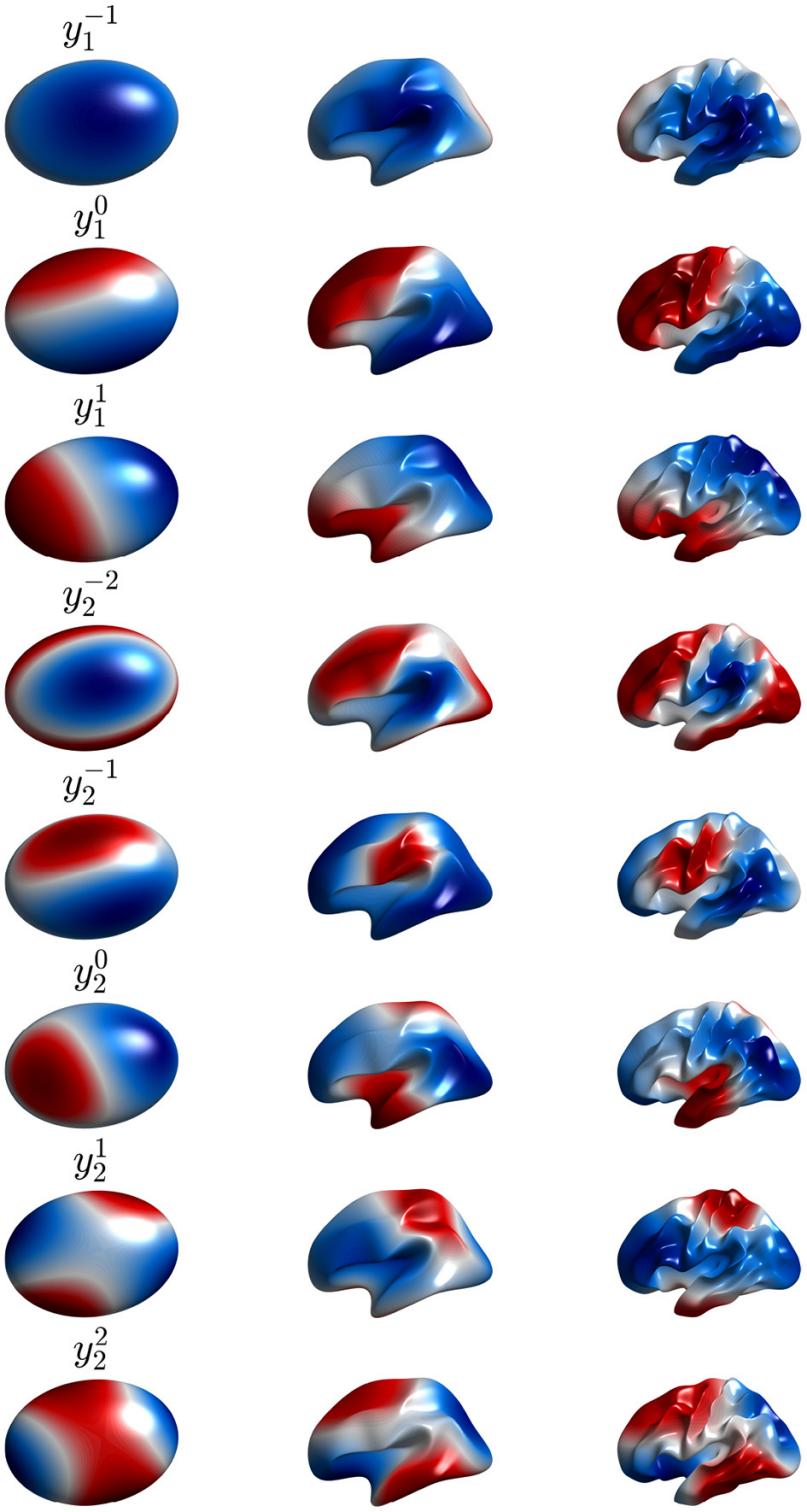
Second to ninth real eigenmodes vs. cortical folding, with each row corresponding to one mode, as labeled. Moving from left to right corresponds to the cortex being gradually folded toward its final shape, starting from an ellipsoid. In each case the modes are calculated on the surface using the formulas of Gabay and Robinson (2017) with positive and negative regions distinguished by color.

Figure 8 shows the curvature on one cortical hemisphere plotted as the ratio of mean curvature κ_*O*_ of an average cortical template surface (Glasser et al., 2016), which is somewhat less folded than an actual individual cortex, vs. the curvature κ_*s*_ = 0.148 cm^−1^ of a sphere of equal area, where the curvature is defined to be the mean of the two principal curvatures at each point (i.e., the mean of the reciprocals of the two principal radii of curvature). Despite the large local values of this ratio, and hence of the Laplacian, the perturbation analysis gave excellent results and showed that the form of the lowest modes is locked in by the relatively mild curvature perturbations due to the gross shape of the brain, as seen in Figure 9, rather than by the more severe local perturbations due to highly curved sulci and gyri (Gabay and Robinson, 2017). The reason for this can be seen from the structure of the perturbation terms that arise in the calculations, as we discuss in the next paragraphs.

If we expand perturbations of ∇^2^ from its spherical form as sums of *Y*_*lm*_ in the spherical coordinates Ω = (ϑ, φ) defined by the Freesurfer mapping, we can write the perturbation as (Gabay and Robinson, 2017).

(55) $$\begin{array}{l} \nabla_{\text{pert}}^{2}\left(\Omega \right)=\underset{l^{\prime }m^{\prime }}{\sum }a_{l^{\prime }m^{\prime }}Y_{l^{\prime }m^{\prime }}\left(\Omega \right), \end{array}$$

where the $a_{l^{\prime }m^{\prime }}$ are angular differential operators; resulting perturbations to the eigenvalues $k_{lm}^{2}$ and eigenfunctions *Y*_*lm*_ involve sums over terms with coefficients proportional to Gabay and Robinson (2017) and Schiff (1968).

(56) $$\begin{array}{l} \int Y_{l_{1}m_{1}}^{*}\left(\Omega \right)Y_{l^{\prime }m^{\prime }}\left(\Omega \right)Y_{lm}\left(\Omega \right)d\Omega , \end{array}$$

which determine the contribution of mode *Y*_*l*_1_*m*_1__ to the perturbed state [see Equations (41, 42) of Gabay and Robinson, 2017 for details]. This integral is related to the Clebsch-Gordan coefficients of quantum theory (Schiff, 1968; Maximon, 2010); for it to be nonzero, perturbations must satisfy

(57) $$\begin{array}{l} m^{\prime }=m_{1}-m, \end{array}$$

(58) $$\begin{array}{l} |l_{1}-l|\leq l^{\prime }\leq l_{1}+l \end{array}$$

This means that only perturbations that satisfy (Equations 57, 58) can affect the eigenfunctions or their eigenvalues. Such selection rules are familiar from conservation of angular momentum in quantum mechanics (Schiff, 1968) and mean that the perturbed *lm* mode can only involve a contribution from *Y*_*l*_1_*m*_1__ if perturbations have a component $Y_{l^{\prime }m^{\prime }}$ that satisfies Equations (57, 58). Hence, because activity and connectivity patterns are dominated by low-order modes with *l* ≲ 3, only low-order perturbations can perturb them significantly. Indeed, it has been found that the dominant changes to low-order modes are contributed by modes with the same *l* (Robinson et al., 2016; Gabay and Robinson, 2017), which corresponds to coordinate rotation in the spherical case. Figure 7 shows the first nine real eigenmodes calculated on the convoluted cortex.

In Gabay and Robinson (2017) it was shown that eigenfunctions with small *l* and *m* in the spherical limit are pinned to specific orientations by large-scale curvature of the cortex on similar ~10 cm scales to the spatial structure of the eigenfunctions themselves, whereas the strong, short-scale curvature due to gyri and sulci on scales of ~2 cm had little effect, despite being larger in amplitude; this is consistent with the arguments in the previous paragraph. Departures from the mean spherical value are large, as seen in Figure 9, but the perturbation matrix element remains small because of the mismatch of spatial scales (Gabay and Robinson, 2017). The circumference of one brain hemisphere is ~60 cm and the $Y_{l}^{m}$ have *l* “wavelengths” (i.e., the typical separation of successive maximums) in this distance, so a 2 cm scale corresponds to *l* ≈ 15 if it represents a half-wavelength. The lack of effect reflects the selection rules discussed in the previous paragraph and that the strong local curvature of sulci and gyri contributes only slightly to low-order modes of the perturbation.

The number of spherical eigenmodes up to and including the level *l* is (*l* + 1)^2^. Hence, to account for the typical numbers of <10 microstates or RSNs, we need to focus on eigenmodes with *l* ≲ 2; activity is also dominated by these modes (Nunez, 1995; Nunez et al., 2001; Robinson et al., 2001, 2016, 2018; Mukta et al., 2017, 2019). Note that when the bihemispheric brain is considered, the number of modes is doubled by including combinations of the present modes that are symmetric and antisymmetric between hemispheres (Robinson et al., 2016).

**Figure 10:**
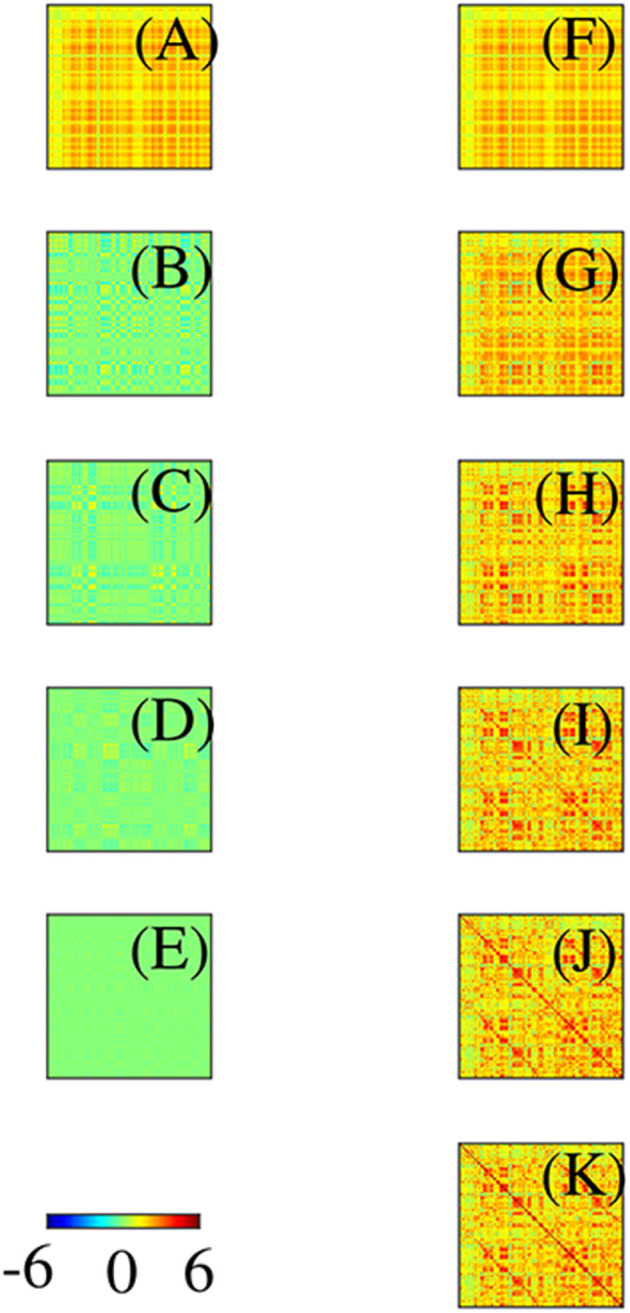
Modal contributions to FC in connection-matrix form, with scale as in the color bar. **(A–E)** Contributions from each of the first 5 modes in order. **(F–J)** Cumulative sums of the frames on the left. **(K)** Exact FC.

Before leaving this section, it is worth stressing that: (i) Even though the eigenmodes on the convoluted cortex are complicated, they can be used perfectly well to expand activity and connectivity via the general formulas in section 2.2, with sums and integrals performed numerically. (ii) The assumption of Laplacian coupling in this section is not necessary; eigenmodes found from measured FC matrices are almost identical because the underlying Green function of propagation is close to the anatomical connectivity (Robinson et al., 1997, 2016; Braitenberg and Schüz, 1998) and analogs of the above analysis in matrix notation are straightforward. We return to this point in section 3. (iii) Activity-based eigenmodes assume only linearity and automatically include the effects of unresolved short-range and subcortical dynamics and connectivity (Robinson, 2019; Gao and Robinson, 2020). (iv) These results imply that low-order modes can compactly represent complex connectivity (Robinson et al., 2016; Gao and Robinson, 2020). For example, Figure 10 shows the contributions to approximation of an experimental Ĉ from the first few modes, and the cumulative approximations obtained by summing them. This demonstrates that as few as 4 or 5 modes can give a good representation of the main features of the FC. One might wonder how the intricate structure, with block-like features, seen in Figure 10 can arise from just a few, smooth, large-scale modes. The reason is that most of the visual appearance of intricacy is an artifact of the mapping of the 2D cortex onto a 1D list of cortical locations (Henderson and Robinson, 2011). (v) Comparison of Figures 10B,C shows that these two modes are almost complements of one another. Hence, if the relative instantaneous amplitudes of activity in these modes were to change so that first one dominated then the other, the FC structure would shift substantially, leading to “switching” behavior when mapped to the instantaneous nearest of a finite set of patterns (Hansen et al., 2015).

It is worth noting that eigenfunctions are not direct equivalents of microstates or RSNs. Microstates are scalp projections of cortical activity, filtered through overlying tissues, so they result from activity in superpositions of multiple eigenstates (Nunez, 1995; Gabay et al., 2018). Likewise, if RSNs are defined to be spatially nonoverlapping with sharp edges and are decomposed into eigenmodes without fully taking into account the processing steps that yield them and the properties of spectral transform (Atasoy et al., 2016), they have a broad eigenspectrum due to their sharp boundaries rather than intrinsic structure in the dynamics. Hence, recent claims to have “explained” RSNs with eigenmodes (Atasoy et al., 2016, 2018) are incorrect *per se*, and trivial if all that is meant is that it is possible to expand RSNs in a series of eigenmodes of a structural connectivity matrix—this is mathematically guaranteed because these eigenmodes form a complete orthonormal basis that can be used to expand any well-behaved function (Schiff, 1968; Courant and Hilbert, 2004).

### 2.4. Control Systems Links

If eigenvalues are distinguished by a subscript *j*, we can approximate the part of *T* that arises from the *j*th mode as Babaie-Janvier and Robinson (2018), Babaie-Janvier and Robinson (2019), and Ogata and Yang (1970).

(59) $$\begin{array}{l} T\left(k_{j},\omega \right)=\underset{p}{\sum }\frac{r_{jp}}{\omega -\omega_{jp}}, \end{array}$$

(60) $$\begin{array}{l} \omega_{jp}=\Omega_{jp}-i\gamma_{jp}, \end{array}$$

where Ω_*jp*_ is the real part of the frequency of pole *jp*, γ_*jp*_ is its damping rate, and *r*_*jp*_ is its weight in the expansion. The reality condition on the Fourier transform implies that any pole with nonzero Ω_*jp*_ must be paired with one at −Ω_*jp*_ with the same damping rate, and a weight that is the complex conjugate of *r*_*jp*_.

The form (59) can be interpreted as a sum of PID (proportional-integral-derivative) filters (Ogata and Yang, 1970; Babaie-Janvier and Robinson, 2018), one at each pole, having the standard control-system roles of implementing prediction and attention via gain control (Babaie-Janvier and Robinson, 2018, 2019, 2020). Each pole corresponds to a resonance in the response and an enhancement in the EEG spectrum (Babaie-Janvier and Robinson, 2018, 2019, 2020), although these only yield distinguishable spectral peaks if the damping is small.

Many authors have noted similarities in the patterns of activity and connectivity observed at different timescales, ranging from the ≲ 0.1 Hz of fMRI to tens of Hz in EEG (Honey et al., 2007; Mantini et al., 2007; Ghosh et al., 2008; Van De Ville et al., 2010; Hipp et al., 2012; Marzetti et al., 2013; Gohel and Biswal, 2015; Vidaurre et al., 2018; Hunyadi et al., 2019). This is easily explained by the fact that activity at all frequencies has the same spatial eigenmodes (Robinson et al., 2016). Corticothalamic NFT has previously explained how multiple frequency resonances exist for each mode (unlike in a violin string, where there is only one frequency per mode) due to the effects of delays in loops between cortex and thalamus (Robinson et al., 2002, 2016; Deco et al., 2008; Gabay and Robinson, 2017). Likewise, the connection between weakly damped resonances and long-range correlations is a straightforward consequence of the physics—weakly damped waves are easily excited and propagate long distances (Robinson, 2003), plausibly supporting long-range in-phase communication via the communication-through-coherence hypothesis (Fries, 2005).

In applications to date it has been found that in the adult human corticothalamic system the main poles have frequencies in the traditional slow/delta, alpha, and beta bands at around 0, 10, and 20 Hz, respectively (Gabay et al., 2018). However, different modes have frequencies differing by ~1 Hz in the alpha band, for example. This means that the modes can beat with one another, giving rise to linear and circular polarization dynamics and directly explaining a number of features and selection effects in measurements of EEG microstates (Gabay et al., 2018) without needing the nonlinear mechanisms invoked by some authors (Roberts et al., 2019).

## 3. Results

In the previous section we reviewed and adapted the background theory and key selection rules for perturbations to affect eigenmodes. In particular, we noted that some connectivity changes can be viewed as responses to stimuli and can thus be absorbed into Λ and *T* (Rennie et al., 1999; Robinson and Roy, 2015; Babaie-Janvier and Robinson, 2019, 2020). We also showed that even strong perturbations that do not satisfy selection rules analogous to Equations (57, 58) do not significantly affect eigenmodes. In the present section we thus use the eigenmodes of the long-term average FC as a robust basis for expanding all dynamical quantities, using real eigenmodes without loss of generality. We then argue that these spatial modes can only be very slightly perturbed by short-term brain state changes because they are strongly pinned by overall brain curvature and any other permanent spatial parameter variations that are present, as seen in Figure 8, and because the selection rules (Equations 57, 58) prevent significant perturbations by local effects; so amplitude changes of activity in these modes are expected to dominate FC dynamics. Finally, we systematically analyze short-term dynamics by expansion in terms of the robust long-term eigenmodes.

Having laid this groundwork, we now turn our attention to treating the effects of dynamic changes in brain EC that cannot be captured as stimulus responses via equations such as Equation (16) and which are not part of the long-term (typically hours or more) structure of the brain. These putative changes have been argued to be responsible for at least part of the observed temporal evolution of FC on timescales from tens to hundreds of seconds, as observed via fMRI (Hindriks et al., 2016; Bassett et al., 2017). We do not concern ourselves here with the important and complex issues of removal of artifacts from signals—e.g., those due to head motion, breathing, and heartbeat for fMRI, and electrical interference for EEG, except to note that we assume that we start with the signals that stem from neural activity, including any global component, which will have its largest projection into the lowest mode. If the latter mode is removed, as is commonly done (examples can be found in Sporns, 2011; Fornito et al., 2016), the FC matrix will tend to develop more negative entries, as has been widely observed, because the lowest mode is the only one without negative correlations between points.

The most common approach to tracking changes in FC is to calculate covariances of temporally windowed fMRI time series. As noted in the introduction, windowed covariances have long been known to give rise to artifacts on timescales of the order of the window length (Yule, 1926; Ernst et al., 2017). The natural bandwidth (Δ*f* ~ 0.07 Hz) of spontaneous fluctuations in fMRI signals, even with static EC, also gives rise to time variations in correlations on a timescale of ~15 s (Pang and Robinson, 2019). When trying to distinguish the effects of true EC changes we must allow for these complications and also set aside evoked changes on time scales of ≲ 15 s, which accords with observations of correlation times and simulations (Zalesky and Breakspear, 2015; Preti et al., 2017).

Numerous methods, discussed in the Introduction, have been proposed to try to deal with the above issues. Many are based on blind signal analysis and/or statistics, while others are ad hoc and often threshold and sparsify data in the interests of simplicity, but without clear justification; few have any regard to the physical nature of the system that generates the signals that are correlated to generate the FC. In contrast, the discussion of eigenmodes in section 2 shows that the physical nature of the dynamics cannot be disregarded without sundering dynamical links that one needs to retain, as illustrated schematically in Figure 1.

Many of the problems with current methods derive from the intrinsic loss of time resolution imposed by windowing—shorter windows are more affected by random variations in noise, whereas long windows lose time resolution. This is a fundamental limit imposed by Nyquist's theorem or the equivalent uncertainty principle and cannot be circumvented by any method. However, the relative effects can be estimated and one can seek to distinguish and minimize averaging effects in a systematic way.

### 3.1. Long-Term Eigenmodes

Stable eigenmodes that are robust with respect to transient system fluctuations are advantageous as the basis for spectral analysis. Ideally, these should be based on activity measures, such as the covariance, to ensure that the effects of spatially unresolved dynamics and connectivity are included, but estimation of these modes can be affected by noise. Modes based on solution of the Helmholtz equation on the cortical surface have also been shown to be a reasonable approximation, and are not affected by noise, but do not incorporate short-range effects nor spatial variations in parameters other than the curvature. We illustrate both types here but do not address experimental data processing issues.

fMRI signals reflect neural activity that drives changes in blood flow and oxygenation. The hemodynamics of these processes restricts the spectrum of these signals to ≲ 0.1 Hz, with a plateau below about 0.07 Hz, where most of the energy is located (Jezzard et al., 2001; Robinson et al., 2006; Drysdale et al., 2010; Pang and Robinson, 2019). Hence, if we wish to calculate mean FC in which the effects of these fluctuations on ~30-s timescales are averaged out, we need to use a window of length *t*_*L*_ with *t*_*L*_ ≳ 300 s, but not so great that state changes due to drowsiness or falling asleep occur, because these can give rise to significant alterations in FC (Tagliazucchi and Laufs, 2014), presumably due to changes in relative mode amplitudes.

Because signals are measured on an array of measurement points (e.g., voxels or discretized regions of interest), we use matrix notation for the covariance matrix and resulting connectivities. But we stress that the discretization must be sufficiently fine to resolve phenomena of interest and reiterate that one must not confuse the structure of the discrete array of measurement points with that of a (nonexistent) discrete macroscale cortical network.

Having calculated the normalized covariance matrix $\bar{C}$ by approximating Equation (3) for τ = 0 by truncating the integral to a long window of duration *t*_*L*_, we obtain its orthonormal eigenfunctions ${\bar{u}}_{j}$ by standard matrix methods. For symmetric connectivity we can obtain $\bar{T}$ and $\bar{\Lambda }$ from Equations (29–31). More generally, even in the asymmetric case, spectral factorization methods can be used to calculate the corresponding nonorthonormal ${\bar{v}}_{j}$ in long-term averaged expressions analogous to Equations (17, 21) if the full correlation function is retained.

**Figure 11:**
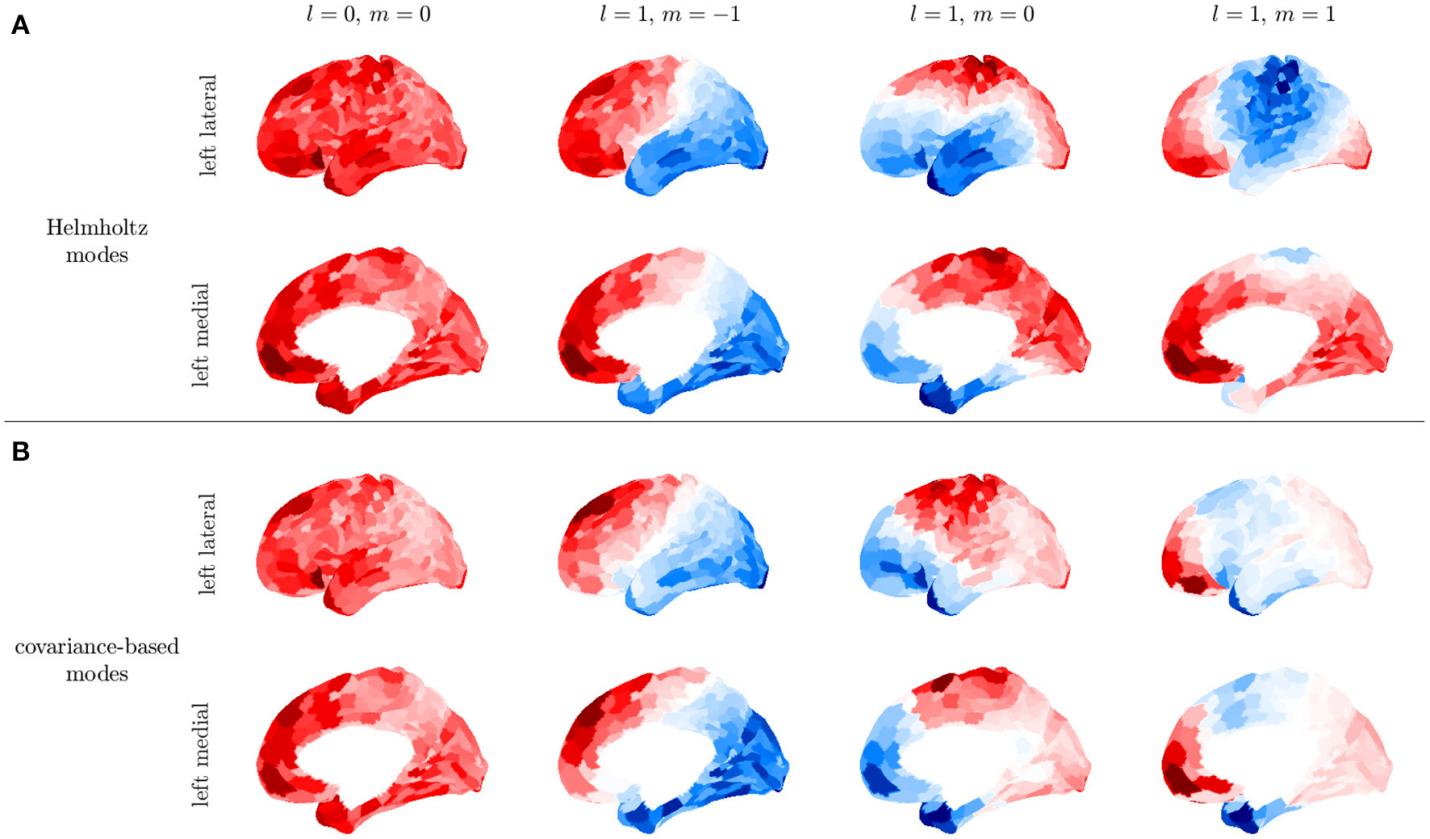
First four modes for the left hemisphere. **(A)** Modes obtained by solving the Helmholtz equation on the cortical surface. **(B)** Covariance-based modes obtained from the average of $\bar{C}$ over subjects in the HCP1200 dataset; for each subject $\bar{C}$ was calculated for a 14 min recording.

We demonstrate the above steps by using the Human Connectome Project HCP 1200 resting fMRI dataset (Glasser et al., 2013). We use postprocessed resting-state fMRI timeseries that have passed through a data processing pipeline to register the measurements onto a standard surface (Glasser et al., 2013) and denoise the data (Glasser et al., 2016) using ICA-FIX. From this release of the data, we remove subjects whose data exhibited incomplete artifact removal as identified in Elam (2019), which yielded a total of 932 subjects. Data are parcellated using the 1000 ROI Schaefer parcellation (Schaefer et al., 2018). To further reduce noise and the effects of incomplete artfact removal, we average *C* over all subject resting state recordings and all 932 subjects. Averaging over individuals restricts us to analyzing low order modes whose spatial variations are larger than the sizes of differences between brains of individuals and therefore relatively unaffected by this averaging compared to high order modes, but this suffices for the present illustrative purposes. Figure 11 shows that the first four modes obtained from this subject averaged fMRI $\bar{C}$ are consistent with the first four modes obtained by solving the Helmholtz equation on the curved cortical surface in section 2.3.3. These low-order $\bar{C}$ modes are obtained purely from data, thus confirming that at least the lowest-order surface-based modes are good approximations, although noise effects on the activity-based modes increase at higher mode numbers. It was previously shown that the lowest order modes were also very similar to ones based on the structural connectivity (Robinson et al., 2016), a fact that follows ultimately from the close similarity of the white matter distribution to the kernel of the Laplacian operator when written in integral form (Robinson et al., 1997). Indeed, the indexes *lm* of the *Y*_*lm*_ can be used to label the corresponding modes on the convoluted cortex.

Commonly discussed fMRI RSNs and EEG microstates have typical linear scales of many cm, so their decomposition must be dominated by low-order eigenmodes. As shown in Figure 8, such modes are spatially pinned by the large-scale curvature of the cortex and even strong localized perturbations do not change them significantly. Moreover, the large-scale curvature produces eigenvalue perturbations that are tens of per cent (Robinson et al., 2016; Gabay and Robinson, 2017). Hence, only large-scale perturbations of other parameters that satisfy selection rules analogous to Equations (57, 58) and have an amplitude large enough to cause similar-sized shifts could potentially change the modal structure significantly. However, early stages of sensory processing are transient and localized to specific areas of cortex, so associated changes will leave large-scale modal structure almost unchanged, but can be expected to alter the relative activity levels in these modes, which will also change the FC; such changes can also result from spontaneous background neural activity.

### 3.2. Instantaneous Activity and Connectivity Kernels

If we use long-term real eigenfunctions ${\bar{u}}_{j}$ to decompose activity as

(61) $$\begin{array}{l} Q\left(\text{r},t\right)=\underset{j}{\sum }c_{j}\left(t\right){\bar{u}}_{j}\left(\text{r}\right), \end{array}$$

(62) $$\begin{array}{l} c_{j}\left(t\right)=\int {\bar{u}}_{j}\left(\text{r}\right)Q\left(\text{r},t\right)d^{2}\text{r}, \end{array}$$

where the integral is over the cortical surface and the ${\bar{u}}_{j}$ are constructed to be real. In matrix notation, these equations become

(63) $$\begin{array}{l} Q\left(t\right)=\underset{j}{\sum }c_{j}\left(t\right){\bar{u}}_{j}, \end{array}$$

(64) $$\begin{array}{l} c_{j}\left(t\right)={\bar{u}}_{j}^{T}Q\left(t\right), \end{array}$$

where the superscript *T* denotes the transpose. Note that Equations (62, 64) are evaluated by integrating over space at a fixed time without temporal averaging.

**Figure 12:**
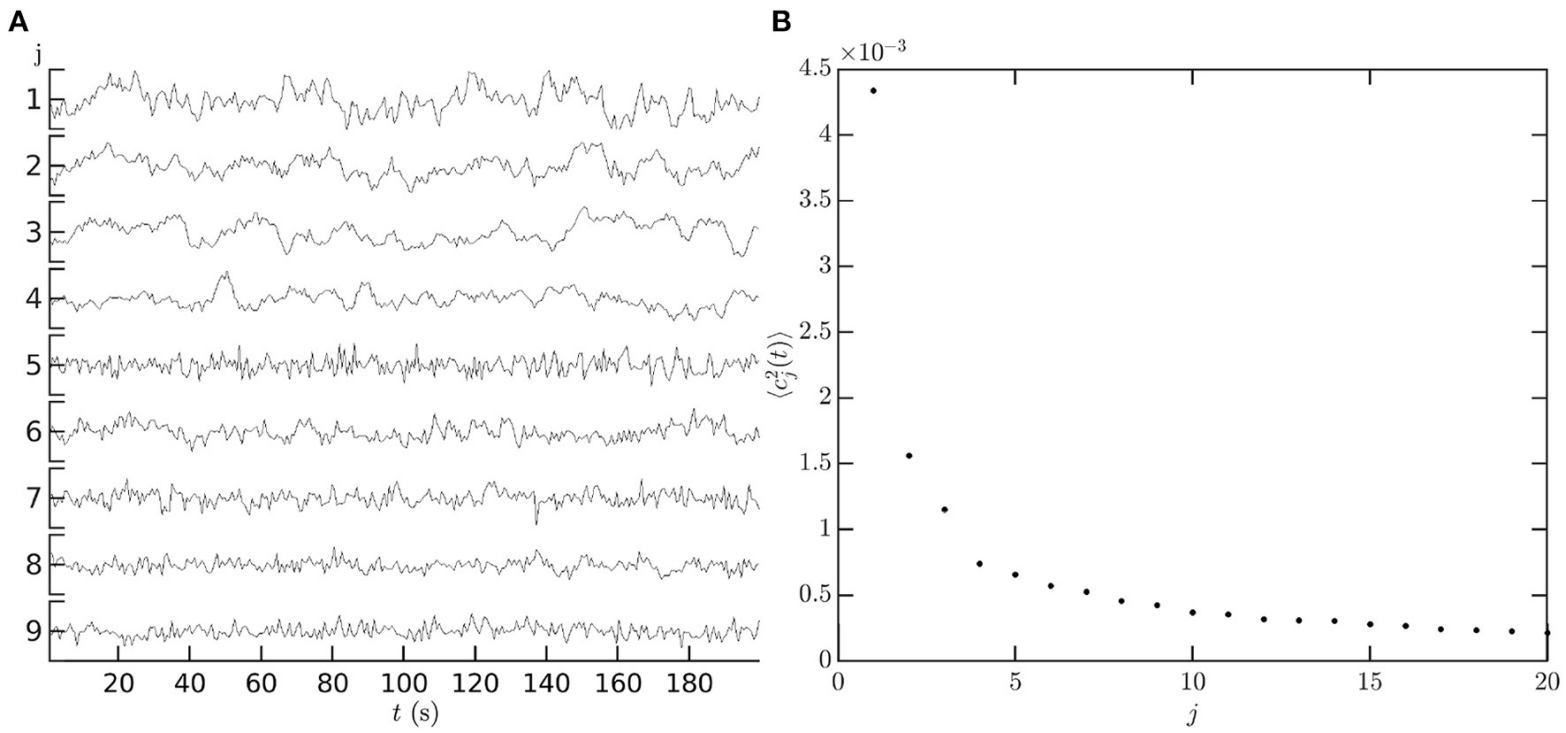
Temporal dependence of mode coefficients. **(A)** *c*_*j*_(*t*), for the first nine covariance modes from a single resting state fMRI scan in the HCP1200 dataset (Glasser et al., 2013). For each mode *j* vertical axis tick marks are at −0.1, 0, 0.1. **(B)** The power of each covariance mode $\langle c_{j}{\left(t\right)}^{2}\rangle$.

Figure 12A shows an illustrative example of the temporal coefficients *c*_*j*_(*t*) of covariance modes obtained from measurements. The first nine left-hemisphere covariance matrix mode coefficients, *c*_*j*_(*t*), are shown for a single subject, single resting state fMRI scan. Data are from the Human Connectome Project 1200 Subject Database (Glasser et al., 2013). The vertex level data are de-meaned and normalized by the mean before being parcellated into 1000 ROIs using the Schaefer 17 network parcellation (Schaefer et al., 2018). The covariance matrix is computed from the ROI timeseries using Equation (3) with τ = 0, and its eigenvectors are then calculated using standard matrix methods (e.g., MatLab's eig function) to give the spatial component of the covariance modes. The spatial components of the covariance modes are then projected back onto the ROI timeseries using Equation (64) to give the mode coefficients *c*_*j*_(*t*). Figure 12B shows that the low order coefficients dominate the power spectrum of the measurements; relatively few modes are required to capture most of the observed dynamics, in comparison to using an ROI or vertex basis of hundreds or thousands of coefficients, respectively.

Setting aside the integral over *t*, the instantaneous kernel of the correlation function in Equation (3) can now be written as

(65) $$\begin{array}{l} \tilde{C}\left(\text{r},t+\tau ;{\text{r}}^{\prime },t\right)=\underset{j}{\sum }{\bar{u}}_{j}\left(\text{r}\right){\bar{u}}_{j}\left({\text{r}}^{\prime }\right)\kappa_{j}\left(\tau ,t\right), \end{array}$$

(66) $$\begin{array}{l} \kappa_{j}\left(\tau ,t\right)=c_{j}\left(t+\tau \right)c_{j}\left(t\right). \end{array}$$

This result decomposes the kernel into a sum of terms that correspond directly to the eigenmodes and whose spatial dependence is evaluated without averaging over multiple temporal measurements. Because the evaluation of the temporal coefficients *c*_*j*_ via Equation (64) involves summation over many spatial points in the discrete approximation, noise is reduced without blurring the signals in time. For example, in fMRI there can be ~ 10^4^ voxels, so noise is reduced by about a factor of 100 relative to the single-voxel value at any fixed *t*, whereas obtaining the same reduction by using ~ 10^4^ temporal measurements would typically take hours, completely average out any short-term dynamics, and almost certainly involve connectivity changes due to drowsiness (Tagliazucchi and Laufs, 2014). If necessary, it is still possible to smooth or interpolate the *c*_*j*_(*t*) over a small number of time points by fitting a moving average, for example, to get an improved estimate at any given time, but we do not use this step in the present work. If robust ${\bar{u}}_{j}$ are available, the above procedure directly projects out the dominant, large-scale modes of interest in studying resting-state fMRI and EEG activity, and suppresses fine-scale spatial features (of noise and activity) in the process, leaving them in the higher modes. This is far more straightforward and mathematically justified than trying to obtain such patterns directly from correlation matrices of voxel- or ROI-level time series that are then thresholded, clustered, and/or sparsified to reduce complexity, and time-averaged to suppress noise.

We can also define the symmetric kernel

(67) $$\begin{array}{l} \kappa_{j}^{\prime }\left(\tau ,t\right)=c_{j}\left(t+\tau /2\right)c_{j}\left(t-\tau /2\right), \end{array}$$

which enables us to define the analogs *C*(τ, *t*) and *K*(τ, *t*) of *C*(τ) and *K*(τ) in Equations (31, 37); covariance corresponds to τ = 0. Equation (67) can be Fourier transformed with respect to τ to obtain *K*(ω, *t*), the ω-dependent kernel centered at time *t*, and thence, via the analogs of Equations (29–33), the ECs *T*(ω, *t, t*_*F*_) and Λ(ω, *t, t*_*F*_), where these Fourier-space quantities necessarily involve integration over a temporal window of width *t*_*F*_ to accomplish the Fourier transform. Hence, there is an unavoidable trade-off between spectral and temporal resolution, dictated by the uncertainty principle, and κ_*j*_(τ, *t*) will vary with *t* due to time-varying activity, even for a static brain structure. Similar results can also be defined for wavelet transforms.

It is also possible to average κ_*j*_(τ, *t*) over a range Δ*t*_0_ of *t*_0_; i.e.,

(68) $$\begin{array}{l} {\bar{\kappa }}_{j}\left(\tau ,t,\Delta t\right)=\int_{-\infty }^{\infty }\kappa_{j}\left(\tau ,t+t^{\prime }\right)w\left(t^{\prime },\Delta t\right)dt^{\prime }, \end{array}$$

where *w*(*t*, Δ*t*) is a weight function with unit integral and a characteristic width Δ*t* in time. In the limit that Δ*t* → ∞, ${\bar{\kappa }}_{j}$ approaches its long-term average and the equations in section 2.2 can be used without modification, while at shorter times, we see from the definition (67) that κ_*j*_ is second order in the amplitude *c*_*j*_ and thus varies on the same timescale as the correlation time (i.e., inverse bandwidth) of the activity. Correlations of the covariances measured at different times are sometimes used as a measure of dynamic FC; however, such correlations are fourth order in the amplitudes, so it cannot have a longer correlation time than the usual second-order activity correlation.

Overall, it is worth stressing that all covariance-based connectivity measures (and by analogy, ones based on other quantities such as coherence) will vary over time due to the nonzero bandwidth of the activity from which they are computed, even if the actual brain effective connectivity is static. We return to this point below.

### 3.3. Direct Determination of *T*

Having obtained the ${\bar{u}}_{j}$ from the covariance, for example, one can construct *T* in an alternate way that only uses data from time intervals of order 1 s, and then use it to obtain $\hat{\Lambda }$. The fact that *T* is by definition the response to a delta function stimulus implies that the response *T*(**r**, **r**_0_, τ) to a unit delta-function stimulus at **r**_0_ and *t* = 0 is given by Equation (2) with $N\left({\text{r}}^{\prime },t\right)=\delta^{2}\left({\text{r}}^{\prime }-{\text{r}}_{0}\right)\delta \left(t\right)$, which yields

(69) $$\begin{array}{l} \theta_{j}\left(\tau \right)=\frac{1}{{\bar{u}}_{j}\left({\text{r}}_{0}\right)}\int {\bar{u}}_{j}\left(\text{r}\right)T\left(\text{r},{\text{r}}_{0},\tau \right)d^{2}\text{r}. \end{array}$$

If the eigenmodes are determined via a high resolution method such as fMRI, or by solutions of the Helmholtz equation, Equation (69) then enables the coefficients θ_*j*_(τ) in Equation (36) to be determined by EEG with good temporal resolution once *T*(**r**, **r**_0_, τ) has been measured at a number of points. Significantly, this does not require high EEG spatial resolution because, by analogy to Fourier transforms, the lowest *M*/2 of the θ_*j*_ can be determined by sampling at *M* points, labeled *k*. One finds (Robinson, 2013; Robinson et al., 2018; Henderson et al., 2021).

(70) $$\begin{array}{l} a_{k}\left(t\right)=T\left({\text{r}}_{k},{\text{r}}_{0},\tau \right), \end{array}$$

(71) $$\begin{array}{l} \approx \overset{M}{\underset{k=1}{\sum }}{\bar{u}}_{j}\left({\text{r}}_{k}\right){\bar{u}}_{j}\left({\text{r}}_{0}\right)\theta_{j}\left(\tau \right), \end{array}$$

(72) $$\begin{array}{l} =\overset{M}{\underset{k=1}{\sum }}b_{kj}\theta_{j}\left(\tau \right), \end{array}$$

(73) $$\begin{array}{l} b_{kj}={\bar{u}}_{j}\left({\text{r}}_{k}\right){\bar{u}}_{j}\left({\text{r}}_{0}\right), \end{array}$$

where Equation (70) defines *a*_*k*_(*t*) and Equation (36) has been used in obtaining Equation (71). Equation (72) can be inverted to obtain the θ_*j*_(τ), and thence *T*, so long as the condition number of the matrix of the *b*_*kj*_ is not too large, which would correspond to a measurement or stimulus point lying very near a zero of one of the modes. The simplest solution in such a case is to add or substitute more measurement points, which would also reduce the effects of noise.

This procedure enables fusion of high spatial resolution fMRI data with high temporal resolution ERP time series (see Figure 1A) via a sparse representation of modes that is analogous to the use of amplitude and phase to describe Fourier modes (Robinson, 2013). It does employ long-term eigenmodes, but does not involve any other windowing of the data, and can probe rapid changes in response via standard ERP protocols (Luck and Kappenman, 2012). In principle, by allowing sparse nonuniform sampling, it also solves the problem of the relatively coarse-grained resolution of EEG measurements and their inability to probe activity in sulci (Nunez, 1995).

### 3.4. Dynamic FC

Functional connectivity is most commonly defined to be the two-point covariance of signals, often normalized to constant variance and evaluated using fMRI averaged over a long window of length *t*_*L*_. However, Yule showed that windowed covariance can exhibit artifactual dynamics even for a pair of sinusoidal signals of fixed relative phase, due to the interaction between the window length and the wave period (Yule, 1926). This analysis has been extended to two signals generated by Wiener processes, where the covariance has been found to be volatile even in a system of fixed structure (Yule, 1926; Ernst et al., 2017), and has also been revisited by Zalesky and Breakspear (2015), Preti et al. (2017), and Leonardi et al. (2015), and has been further clarified above. The problem is to try to separate the covariance changes that occur due to the finite bandwidth of the activity and the window length from those due to underlying EC changes.

#### 3.4.1. Violin String Analog System

Here we analyze a simple example of the dynamics of FC under spontaneous conditions to clarify the roles of bandwidth, mode beating, and transients, and point out intrinsic shortcomings of analysis methods that seek “switching” between patterns from a precompiled dictionary or cluster-based analysis. To do this we use a simple 1D two-mode example to examine the ways in which mode frequencies and bandwidths can affect FC even when there is no change in the underlying EC. All the results here can be straightforwardly generalized to include more modes, continuous frequency spectra, 2D effects, and different boundary conditions.

Let us first consider a 1D brain akin to the violin string in Figure 2, in the absence of damping and regeneration of activity, but now with periodic boundary conditions that identify the left and right boundaries with one another and allow nonzero displacement at all points, including the two points that were fixed in Figure 2. The lowest mode here is uniform in space and *k*_*j*_ = 2π*j*/*L* with *j* = 0, 1, 2, ….

If all the activity is in the lowest two nonuniform modes, and the transfer function has two pairs of poles at slightly different frequencies we have

(74) $$\begin{array}{l} Q\left(x,t\right)=u_{1}\left(x\right)\left[\sin\left(\Omega_{11}t\right)+\sin\left(\Omega_{12}t\right)\right]\\ \;+u_{2}\left(x\right)\left[\sin\left(\Omega_{21}t\right)+\sin\left(\Omega_{22}t\right)\right],\\ \;=2u_{1}\left(x\right)\sin\left(\Omega_{1}t\right)\cos\left(\Delta \omega_{1}t\right) \end{array}$$

(75) $$\begin{array}{l} +2u_{2}\left(x\right)\sin\left(\Omega_{2}t\right)\cos\left(\Delta \omega_{2}t\right), \end{array}$$

(76) $$\begin{array}{l} u_{1}\left(x\right)=\sin\left(2\pi x/L\right), \end{array}$$

(77) $$\begin{array}{l} u_{2}\left(x\right)=\cos\left(2\pi x/L\right), \end{array}$$

(78) $$\begin{array}{l} \Omega_{j}=\left(\Omega_{j1}+\Omega_{j2}\right)/2, \end{array}$$

(79) $$\begin{array}{l} \Delta \omega_{j}=\left(\Omega_{j1}-\Omega_{j2}\right)/2 \end{array}$$

in the simplified case that all poles are of equal strength. Typically, nonzero mode frequencies in the corticothalamic system are of order 3–20 Hz (Babaie-Janvier and Robinson, 2018, 2019, 2020; Gabay et al., 2018) and the frequency difference between poles in the same band is of order 1 Hz or less (Gabay and Robinson, 2017; Gabay et al., 2018). Moreover, multiple modes typically have resonances close to each other in a given band (Gabay et al., 2018). In Equation (74) the mode coefficients vary sinusoidally in time, but if a form analogous to Equation (59) is used, we can separate off the sinusoidal part and fully specify the dynamics by the constant coefficients of these functions, which is even more compact. More generally, these coefficents may vary on timescales longer than the period of the oscillations (if they varied on a shorter timescale, clear oscillations would not be seen).

Equation (75) shows that each mode will display fast oscillations at each nonzero Ω_*j*_ and slow beating at Δω_*j*_. These have been shown to account for properties of EEG microstates, including effects that lead to preferential detection of the particular patterns that occur at the extremums of the beats (Gabay et al., 2018).

If we write the instantaneous contribution of the above activity to the covariance as *c*(**r**, **r**′, *t*, τ = 0) before any temporal averaging, then

(80) $$\begin{array}{l} c\left(\text{r},{\text{r}}^{\prime },t,\tau =0\right)=u_{1}\left(x\right)u_{1}\left(x^{\prime }\right)\sin^{2}\left(\Omega_{1}t\right)\cos^{2}\left(\Delta \omega_{1}t\right)\\ \;+u_{2}\left(x\right)u_{2}\left(x^{\prime }\right)\sin^{2}\left(\Omega_{2}t\right)\cos^{2}\left(\Delta \omega_{2}t\right)\\ \;+\left[u_{1}\left(x\right)u_{2}\left(x^{\prime }\right)+u_{2}\left(x\right)u_{1}\left(x^{\prime }\right)\right]\\ \;\times \sin\left(\Omega_{1}t\right)\sin\left(\Omega_{2}t\right)\cos\left(\Delta \omega_{1}t\right)\cos\left(\Delta \omega_{2}t\right), \end{array}$$

which gives a FC that is manifestly time- and frequency-dependent. Again, if averaging is done over the fast oscillations, only the first two terms on the right remain, modulated by slow oscillations that arise from the beats, and giving

(81) $$\begin{array}{l} c\left(\text{r},{\text{r}}^{\prime },t,\tau =0\right)=\frac{1}{2}u_{1}\left(x\right)u_{1}\left(x^{\prime }\right)\cos^{2}\left(\Delta \omega_{1}t\right)\\ \;+\frac{1}{2}u_{2}\left(x\right)u_{2}\left(x^{\prime }\right)\cos^{2}\left(\Delta \omega_{2}t\right). \end{array}$$

The FC will thus vary on long timescales ~1/|Δω_*j*_|, spending disproportionately large time intervals near one or other extremum of the cos^2^ factors, and switching quasiperiodically between them; however, these extremums are not “metastable” (i.e., in stable local equilibrium but with a small margin of stability relative to other more stable states), contrary to a misleading usage that has crept into some corners of the literature; the term “metastable” should not be used to describe patterns that are merely transient.

Because Δω_1_ ≠ Δω_2_ in general, the two contributions in Equation (76) will gradually drift in and out of phase, so FC estimates on scales of ~ 1 s will vary even without any change in modes, modal structure, or mean activity in the individual modes. Further averaging over the beat timescales would replace the cos^2^ terms by 1/2. Hence, we have three regimes of dynamics and window-length *t*_*L*_ for corresponding averaging: (i) *t*_*L*_ ≫ 2π/|Δω_*j*_| where long-term average FC is recovered; (ii) 2π/Ω_*j*_ ≲ *t*_*L*_ ≲ 2π/|Δω_*j*_| where beats of activity induce FC modulations and interact with the window length to give spurious apparent dynamics; and (iii) *t*_*L*_ ≲ 2π/Ω_*j*_ where fast oscillations modulate FC on timescales detectable by EEG methods.

**Figure 13:**
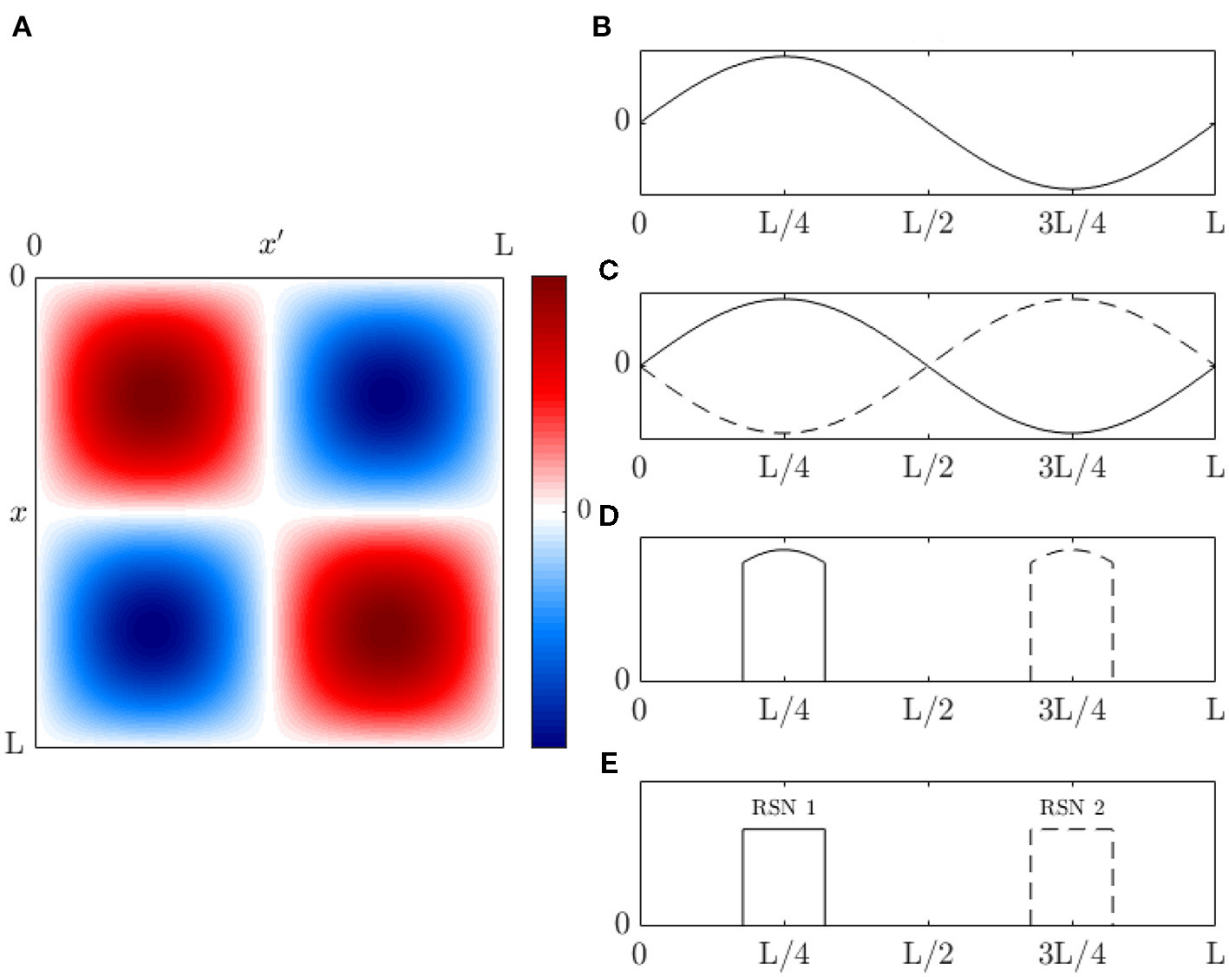
Schematic RSNs of a violin string with periodic boundary conditions. **(A)** Correlations between points at *x* and *x*′ for the mode shown in **(B)**, with positive values shown red and negative blue. **(B)** One sinusoidal mode. **(C)** Correlations with the points *L*/4 (solid) and 3*L*/4 (dashed). **(D)** Thresholded correlations for the two cases in **(C)**. **(E)** RSNs defined by large positive correlations between points.

Turning to the spatial properties of Equations (75, 81), we first note that points where *u*_*j*_(*x*) and $u_{j}\left(x^{\prime }\right)$ have the same sign will be positively correlated, as shown in Figures 13A,B. This means that there will be strong positive correlations near *x* = *L*/4, 3*L*/4 for mode 1, but these two regions will be strongly anticorrelated with one another. Hence, methods that group regions with high positive correlations into RSNs will erroneously split each mode except the lowest into two separate RSNs, as seen in Figures 13C,D, despite it being a single dynamical entity. Seed-based clustering methods that ignore negative correlations will thus tend to return four separate RSNs in place of the two modes in Equation (80). Similar comments apply to methods that cluster spatial points on the basis of similarity of patterns of incoming or outgoing connectivity (Yeo et al., 2011). Many of these methods do find robust patterns of the type they impose, but they break the underlying dynamical links and suffer from thresholding effects, artificial discretization, omission of negative correlations, assumptions of nonoverlapping patterns, and/or other restrictions built into the methods themselves, as discussed in the Introduction.

The temporal variations noted above will cause the peaks and troughs of the covariance to wax and wane on timescales ~ 2π/|Δω_*j*_|. In general, this will cause the two pairs of regions of highly correlated activity to come and go so that 0, 2, or 4 extremums will be detectable above some magnitude threshold, which would normally be set by experimental noise or imposed by fiat to keep the number of patterns small. Methods that rely on matching to a dictionary of predetermined FC patterns will thus appear to show sudden switching between patterns, despite the underlying dynamics being smooth and without jumps. Such apparent transitions are thus a spurious artifact of moment-by-moment matching of continuous evolution to the nearest pattern in a discrete list.

#### 3.4.2. Limitations on Detecting Dynamic FC

The example in the previous subsection can be generalized to cases in which the power spectrum is continuous and many resonances are present at once. In the most relevant case, in which spontaneous activity spectra peak at zero frequency, one simply sets Ω_*j*_ = 0 and replaces the bandwidth Δω_*j*_ by the total bandwidth of the spectrum.

**Figure 14:**
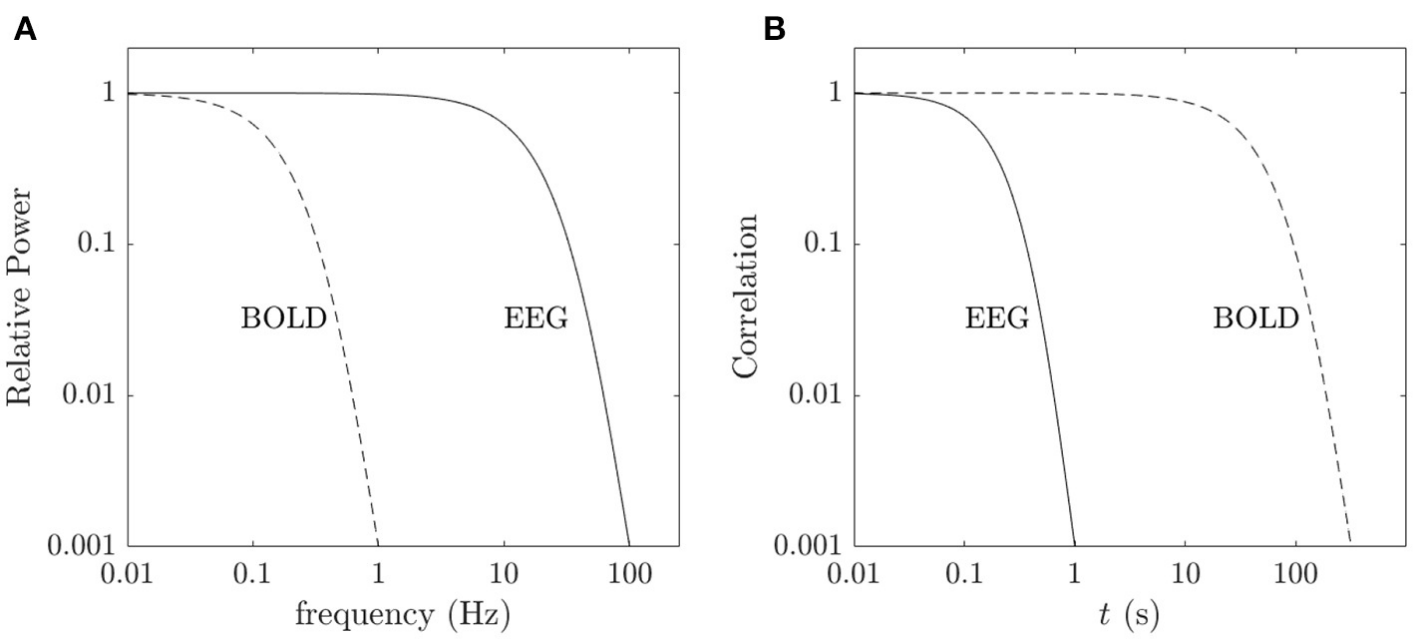
Frequencies and timescales relevant to FC dynamics. **(A)** Schematic EEG (solid) and BOLD (dashed) spectra, with characteristic bandwidths of ~ 15 Hz and ~ 0.1 Hz, respectively. **(B)** Schematic correlation functions of EEG (red) and BOLD (blue) fluctuations, with characteristic correlation times of 0.1 s and 15 s, respectively.

Figure 14 shows schematic spectra and temporal correlation functions of spontaneous fMRI BOLD signals and EEG or MEG signals, normalized to unity at low frequencies. These have bandwidths and correlation times *t*_*c*_ of order Δ*f* ≈ 0.07 s and *t*_*c*_ ≈ 15 s for BOLD and Δ*f* ≈ 15 Hz and *t*_*c*_ ≈ 0.07 s for EEG or MEG, respectively.

Two regimes are defined by the correlation functions in Figure 14B. The first regime corresponds to measurement times much less than *t*_*c*_. In this case activity fluctuations (and hence fluctuations in the FC even for static EC) are essentially static during a given measurement. Here it is possible to obtain a “snapshot” of FC, but this timescale (≪0.2 s) is too short to be useful for EEG or MEG. In the case of fMRI timescales of a few seconds can be probed and such responses can be incorporated into the evoked response, as discussed in section 2.3.1.

The second regime corresponds to measurement times much greater than *t*_*c*_. Here, fluctuations in FC on timescales of order *t*_*c*_ will tend to average out, thereby exposing modifications in FC that are due to EC changes on longer timescales. For fMRI this restricts attention to timescales of a 100 s or more, as has been argued previously (Leonardi et al., 2015; Zalesky and Breakspear, 2015). EEG and MEG methods can reach this regime on the scale of a few seconds or longer. This has the potential to enable EC changes to be monitored on timescales much shorter than those of alertness dynamics, and potentially to be probed by the evoked-response approach in section 3.3.

## 4. Summary and Discussion

We have used eigenfunction-based spectral methods to analyze dynamical brain connectivity in the usual linear regime, drawing together and generalizing many results from spectral analysis, propagator theory, and neural field theory in the process. The results clarify the limitations on a range of ad hoc methods for FC analysis and point the way to avoiding some of their pitfalls, although it is not possible to analyze individually the multitude that have been proposed.

Central to this work is the recognition that the brain is a physical system, understanding of which is facilitated by taking its physical nature into account. This is reflected in our use of system eigenfunctions as the physical building blocks of its dynamics and connectivity, rather than employing statistically constructed “networks.” We also avoid treating the set of links between artificially discretized brain regions as being an actual macroscale brain network—no such network exists. We do acknowledge that some such phenomenological approaches yield measures that are useful for classification and estabishment of case-control distinctions, for example, but they tend to obscure the nature of the underlying dynamics, which is the focus of the present work.

The main findings are:

- Links between activity, EC, and FC have been clarified, including short-term modulation of EC as part of the evoked response to a stimulus (e.g., via gain dynamics) being able to be absorbed into the definition of the transfer function (Babaie-Janvier and Robinson, 2020). The EC-FC connection is fundamental because directly observable structural connectivity (SC) does not capture short-range connectivity, especially of inhibitory neurons, and also says nothing about which neurons are active in a given situation. The SC-EC connection is thus a separate issue.
- Eigenmodes of a violin-string analog system were used as a foil to elucidate the concepts, spatial brain eigenfunctions being analogous to the spatial eigenfunctions that correspond to individual notes from a musical instrument. This analogy has been used to clarify how every eigenmode spans the whole system and every point participates in each eigenmode. This invalidates classes of methods based on thresholding and/or clustering that seek to find discrete patterns (or “networks”) that have sharp boundaries and are mutually exclusive.
- Eigenmode analysis emphasizes how smooth evolution of activity gives rise to time-dependent FC. Beating of modes at different frequencies will give rise to changes in activity and FC patterns without requiring nonlinear mechanisms (Gabay and Robinson, 2017). Resulting dominance of different modes at different times can yield substantial FC changes even when the EC is static. Methods that match instantaneous FC to the nearest entry in a finite dictionary of patterns will produce sudden, but artifactual, “switching” as a result.
- EC and FC are dominated by a moderate number of low-order modes, which explains the robustness of a relatively small number of patterns seen in EEG and fMRI experiments—microstates and resting-state networks, respectively. The dominance of just a few modes also explains the observed similarity of FC at timescales ranging from seconds to tens of minutes.
- There is not a one-to-one mapping between eigenmodes and patterns such as RSNs or microstates. Generally, phenomenological patterns can be decomposed into superpositions of multiple eigenmodes, but the common steps of thresholding and imposition of mutual exclusivity usually lead to the results being noncompact and subject to serious artifact. If such steps are avoided, few-component expansions should be possible, with observed patterns being akin to musical chords, with eigenmodes corresponding to notes.
- Eigenfunctions have been shown to be robust to perturbations except where these are large and of similar spatial scale to variations in the eigenfunction amplitude, as governed by selection rules analogous to those for interactions in quantum physics. Eigenmode robustness explains the general similarity of FC patterns across arousal states. Dynamic changes in FC are thus likely to be dominated by activity changes, rather than changes in EC; for example, deep sleep has enhanced low-frequency activity, which is correlated with lower-order modes and larger correlation lengths and may explain FC differences between arousal states (Nunez, 1995; Robinson, 2003; Chang et al., 2016).
- It has been shown that robust low-order eigenmodes can be estimated from fMRI covariance data, for example, and that these are very similar to modes found by solving the Helmholtz equation on the cortical surface, which further supports their validity. This finding complements earlier work that showed similarities between surface-based modes and ones obtained from structural connectivity (Robinson et al., 2016). Higher-order covariance-based modes are increasingly affected by noise, at least for the data pipeline used in the HCP1200 data set. Denoising is an active area of research that promises to deliver better results in the future, but is beyond the scope of the present aper.
- Projection of activity onto eigenmodes enables direct estimation of their coefficients as functions of time. For activity evoked by an impulse stimulus, this permits calculation of the transfer function, and thus the EC and implied FC, on timescales as short as a second or so using EEG or MEG. This method also has the advantage that multiple presentations under task or background conditions can be used to average out the effects of uncontrolled background activity, as is usual in evoked response experiments.
- Calculation of eigenmode coefficients provides a compact few-mode representation of activity, EC, and the kernels of FC without averaging over time. This reduces noise because short-scale noise predominantly affects high-order spatial modes, removes artifacts due to windowing, and improves time resolution. Tracking mode coefficients vs. time is thus likely to be superior to covariance-based dynamic FC for many purposes.
- Because activity changes contribute to FC dynamics, changing FC is a mandatory consequence of the finite bandwidth of the signals even when EC is fixed, and the window length used to evaluate mean FC compounds this with artifact (Yule, 1926). These effects are not always separable, but the temporal regimes required to probe them have been elucidated, with the correlation time *t*_*c*_ of background activity being the key parameter. For observations on timescales much less than *t*_*c*_, activity fluctuations can be viewed as static, whereas they tend to average out for times much greater than *t*_*c*_, thereby potentially exposing EC changes that are not direct responses to activity.
- As noted above, eigenmodes form a discrete set, with activity dominated by the lowest-order members. Moreover, the frequency response of each mode can be closely approximated by a discrete sum of resonant responses. The latter sum can be truncated to a few terms, each of which represents the dynamics of a PID filter that links the resulting activity to control-systems functions such as prediction and gain-adjustment, the latter implementing a form of attention (Babaie-Janvier and Robinson, 2018, 2019, 2020). These facts imply that eigenmodes form an actual discrete set of entities for the transmission of brain activity, as opposed to “networks” constructed artificially on the basis of arbitrary sensor locations or brain parcellations. The difference here is that the “nodes” are spatially extended eigenmodes; however, these are local in the spectral domain, where they form a discrete lattice labeled by spatial eigenvalues and resonant frequencies.
- Spatial modes plausibly enable spatial communications channels with activity broadcasted into modes, especially near their peaks (Robinson et al., 2018), and available to be read out near other peaks. Significantly, as the number of active modes increases, a steep increase in the number of such channels occurs. Likewise, mode resonances enhance temporal coherence and may thus facilitate a version of the communication through coherence proposal, where in-phase activity can lead to nonlinear enhancement of local firing rates (Fries, 2005).
- Just as in fields such as nonlinear optics and nonlinear plasma theory, linear modes provide the appropriate starting point to analyze such dynamics systematically via perturbation techniques such as mode-coupling expansions (Butcher and Cotter, 1990; Melrose and McPhedran, 1991).

Overall, the above findings reinforce the advantages of using physically based approaches that work with physical objects and respect the underlying dynamics, and analyzing them via standard mathematical methods whose properties and limitations are thoroughly understood. Such approaches avoid working with unphysical objects, failure to respect dimensionality and units, category errors such as attributing discrete properties to continuous systems or quantities, and forced attribution to the brain of characteristics that are actually imposed by the analysis methods used (e.g., discreteness, graph-theoretic properties such as motifs and degree, sharp boundaries, tree-like hierarchies, or modularity, although some of these can be redefined to be valid in the limit of increasingly fine discretization Robinson, 2019). Seductive, but largely unjustified, steps such as sparsification, thresholding, and clustering are also avoided, along with the use of idiosyncratic analyses whose properties and relationships to other approaches are poorly understood. Thus, in keeping with Occam's Razor, new mathematical methods and constructs should be eschewed unless established methods are inadequate.

Systematic methods enable many problems to be attacked that could not otherwise be addressed, and the results to be unified into an overarching framework that spans activity and connectivity. However, the flip-side of this is that one must learn and acquire facility with a range of well-established mathematical and physical techniques—statistical methods alone are not enough to understand dynamics and connectivity beyond phenomenological classification.

## Data Availability Statement

Publicly available datasets were analyzed in this study. This data can be found here: Human connectome project HCP 1200 Subject Resting fMRI dataset, https://www.humanconnectome.org/study/hcp-young-adult.

## Author Contributions

PR conceived the project, carried out the analytic work, and wrote most of the manuscript. JH, KA, and NG contributed to the analysis. JH, KA, NG, TB-J, and XG contributed numerical work embodied in quantitative figures. All authors reviewed the MS and contributed to its final text.

## Conflict of Interest

The authors declare that the research was conducted in the absence of any commercial or financial relationships that could be construed as a potential conflict of interest.
